# Towards data-driven biopsychosocial classification of non-specific chronic low back pain: a pilot study

**DOI:** 10.1038/s41598-023-40245-y

**Published:** 2023-08-12

**Authors:** Scott D. Tagliaferri, Patrick J. Owen, Clint T. Miller, Maia Angelova, Bernadette M. Fitzgibbon, Tim Wilkin, Hugo Masse-Alarie, Jessica Van Oosterwijck, Guy Trudel, David Connell, Anna Taylor, Daniel L. Belavy

**Affiliations:** 1 Institute for Physical Activity and Nutrition, School of Exercise and Nutrition Sciences, Deakin University, Geelong, Australia; 2 Orygen, 35 Poplar Rd, Parkville, VIC 3052 Australia; 3 Centre of Youth Mental Health, University of Melbourne, Melbourne, VIC Australia; 4 Data to Intelligence Research Centre, School of Information Technology, Deakin University, Geelong, Australia; 5 Department of Psychiatry, Faculty of Medicine, Nursing and Health Sciences, Monash University, Melbourne, VIC Australia; 6 Monarch Research Group, Monarch Mental Health Group, Sydney, Australia; 7 Département de Réadaptation, Centre Interdisciplinaire de Recherche en Réadaptation et Integration Sociale (Cirris), Université Laval, Quebec City, Canada; 8 Spine, Head and Pain Research Unit Ghent, Department of Rehabilitation Sciences, Faculty of Medicine and Health Sciences, Ghent University, Ghent, Belgium; 9 Department of Rehabilitation Sciences and Physiotherapy, Faculty of Medicine and Health Sciences, University of Antwerp, Antwerp, Belgium; 10 Research Foundation–Flanders (FWO), Brussels, Belgium; 11 Pain in Motion International Research Group, Brussels, Belgium; 12 Department of Medicine, Division of Physical Medicine and Rehabilitation, University of Ottawa, Ottawa, Canada; 13 Bone and Joint Research Laboratory, Ottawa Hospital Research Institute, Ottawa, Canada; 14 Department of Biochemistry, Microbiology and Immunology, Faculty of Medicine, University of Ottawa, Ottawa, Ottawa, Canada; 15 Imaging@Olympic Park, AAMI Park, 60 Olympic Boulevard, Melbourne, VIC 3004 Australia; 16 Division of Physiotherapy, Department of Applied Health Sciences, Hochschule für Gesundheit (University of Applied Sciences), Gesundheitscampus 6-8, 44801 Bochum, Germany

**Keywords:** Diagnostic markers, Occupational health

## Abstract

The classification of non-specific chronic low back pain (CLBP) according to multidimensional data could guide clinical management; yet recent systematic reviews show this has not been attempted. This was a prospective cross-sectional study of participants with CLBP (n = 21) and age-, sex- and height-matched pain-free controls (n = 21). Nervous system, lumbar spinal tissue and psychosocial factors were collected. Dimensionality reduction was followed by fuzzy c-means clustering to determine sub-groups. Machine learning models (Support Vector Machine, k-Nearest Neighbour, Naïve Bayes and Random Forest) were used to determine the accuracy of classification to sub-groups. The primary analysis showed that four factors (cognitive function, depressive symptoms, general self-efficacy and anxiety symptoms) and two clusters (normal versus impaired psychosocial profiles) optimally classified participants. The error rates in classification models ranged from 4.2 to 14.2% when only CLBP patients were considered and increased to 24.2 to 37.5% when pain-free controls were added. This data-driven pilot study classified participants with CLBP into sub-groups, primarily based on psychosocial factors. This contributes to the literature as it was the first study to evaluate data-driven machine learning CLBP classification based on nervous system, lumbar spinal tissue and psychosocial factors. Future studies with larger sample sizes should validate these findings.

## Introduction

Non-specific chronic low back pain (CLBP) is diagnosed after excluding specific causes of back pain and radicular syndromes, representing ~ 90% of all back pain^1^. The high proportion of low back pain falling into this category prompted the development of classification systems to guide clinical management^2^. Yet, such approaches have demonstrated limited effectiveness, potentially related to: (1) lack of consideration for multidimensional biopsychosocial factors, (2) classification based solely on subjective clinician opinion, (3) the required high skills levels to use them and (4) lack of adequate reliability to be implemented on a widescale^3^. Addressing these issues could improve classification and consequently the personalised care of individuals with CLBP.

Biological, psychological and social factors are associated with CLBP^4^, while the contribution of each domain is likely to differ across individuals^5^. Each of these domains may impact nociceptive pathways and the resulting pain experience^6^. Nervous system factors such as functional connectivity (particularly through the default mode network)^7,8^, grey matter volumes^9^ and sensory tests (pressure-pain thresholds, temporal summation and exercise-induced hypoalgesia)^4^ differ between individuals with CLBP and pain-free controls, suggesting alterations in peripheral and central processing of nociceptive stimuli^6^. Lumbar spinal tissue damage is also more prevalent in individuals with CLBP compared to pain-free controls^10^, which may generate ongoing peripheral nociceptive activity. Psychosocial factors, such as depression and anxiety, can also modulate pain intensity and disability in CLBP^4,6^. Therefore, classification systems for the targeted management of CLBP should consider nervous system, spinal tissues and psychosocial factors. However, a recent systematic review showed that studies do not consider collecting data on all these factors^4^.

Data-driven machine learning classifiers can detect patterns in biopsychosocial data across various pain-related conditions^11^ and could overcome prior limitations with CLBP classification^12^. However, we conducted systematic review that showed only binary classification of low back pain and pain-free controls has been attempted^12^. To follow-up on this, we conducted a data-driven machine learning study and showed accurate CLBP classification using data from the UKBioBank^13^. However, the UKBioBank was lacking data important to CLBP classification, such as spinal tissue factors^13^. This is a problem as the classification of CLBP should be based on all domains associated with the condition. Therefore, the aim of this pilot study was to produce a prospective data-driven classification of CLBP using nervous system, spinal tissue and psychosocial factors. This study contributes to the literature as it was the first study to evaluate data-driven machine learning classification of CLBP based on nervous system, lumbar spinal tissue and psychosocial factors.

## Results

### Demographics and matching

**Figure 1:**
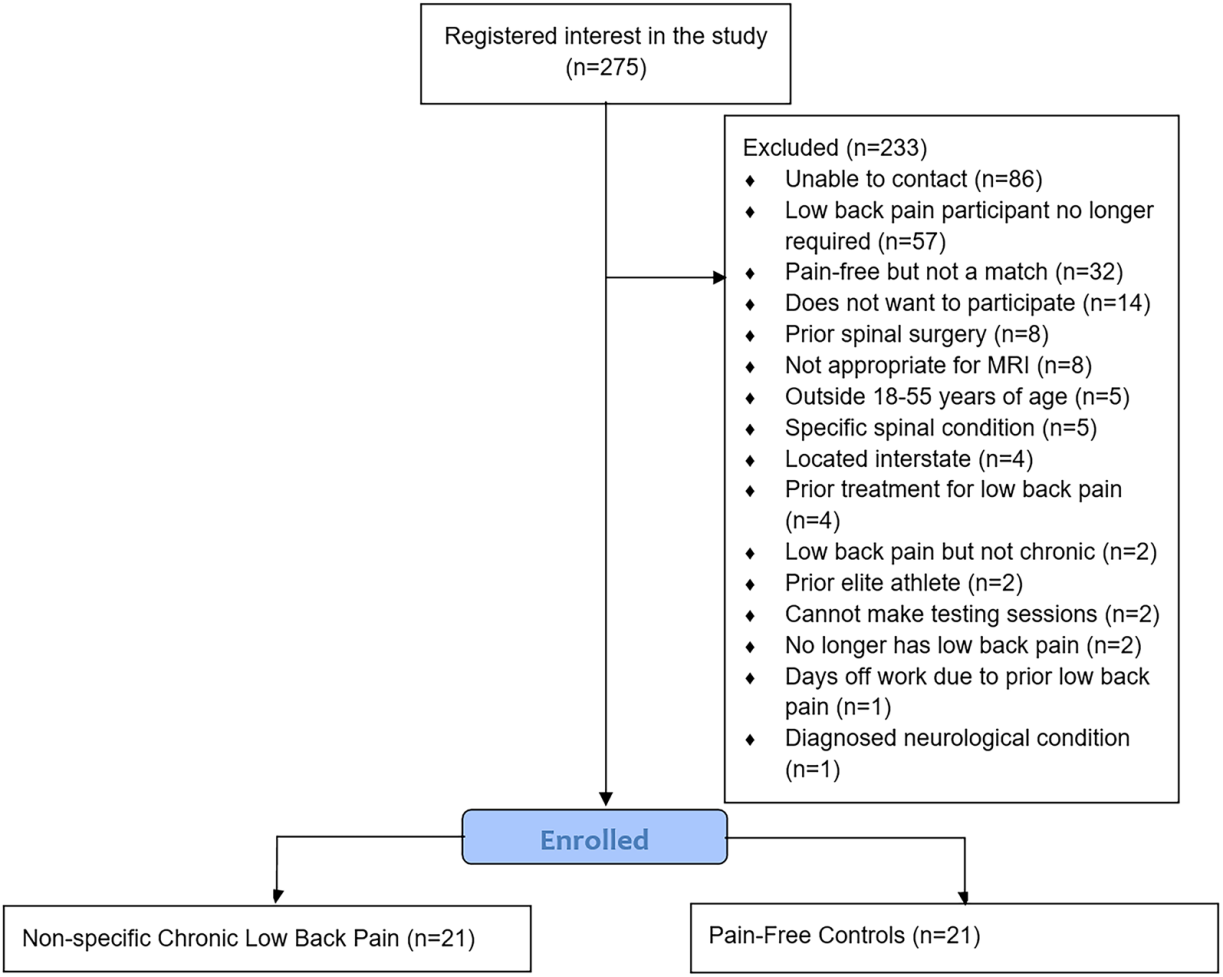
Particant flow diagram.

The participant flow diagram and reasons for exclusion are reported in Fig. 1. Participant characteristics are reported in Table 1. Data for each CLBP participant and the relevant match is available in Supplementary Table 2. After matching participants using self-report height and weight, pain-free controls matched 18/21 (86%) CLBP participants on age, sex, and objectively measured height, and 11/21 (52%) participants on objectively measured body mass index (Supplementary Table 2). The three participants whose controls did not match on height, matched on age, sex and body mass index. No significant demographic differences were observed between the groups (Table 1).

**Table 1:** Participant characteristics.

	Chronic low back pain (n = 21)	Pain-free controls (n = 21)	p
	Mean (SD)	Mean (SD)	
Ages (years)	35.4 (11.0)	35.6 (11.1)	0.956
Sex (male/female—n)	10/11	10/11	–
Height (cm)	174.0 (10.3)	172.9 (9.1)	0.718
Weight (kg)	82.4 (16.9)	75.8 (18.9)	0.238
Body mass index (kg/cm^2^)	27.2 (5.0)	25.3 (6.1)	0.270
Ethnicity n (%)			
Caucasian	17 (81.0)	15 (71.4)	0.304
Asian	2 (9.5)	6 (28.6)	
None of the above	2 (9.5)	–	
Education n (%)			
Year 12	4 (19.0)	1 (4.7)	0.070
TAFE certificate	7 (33.3)	3 (14.3)	
Bachelor degree	4 (19.0)	9 (42.9)	
Postgraduate degree	4 (19.0)	8 (38.1)	
None of the above	2 (9.5)	–	
Employment n (%)			
Unemployed	1 (4.8)	–	0.767
Casual	3 (14.3)	4 (19.0)	
Part-time	5 (23.8)	5 (23.8)	
Full-time	12 (57.1)	12 (57.1)	
Smoking status n (%)			
Current	2 (9.5)	1 (4.7)	0.080
Prior	4 (19.0)	–	
Never	15 (71.4)	20 (95.2)	
Handedness n (%)			
Left	2 (9.5)	4 (19.0)	0.597
Right	17 (81.0)	16 (76.2)	
Ambidextrous	2 (9.5)	1 (4.7)	
Diagnosed depression/anxiety n (%)			
Depression	3 (14.3)	1 (4.7)	0.422
Anxiety	4 (19.0)	3 (14.3)	
Both	4 (19.0)	2 (9.5)	
Neither	10 (47.6)	15 (71.4)	
Pain duration (months)	76.2 (96.2)	–	–
Pain intensity (0–100)			
Current	31.9 (19.8)	–	–
Last-week average	38.4 (17.7)	–	–
Last-week worst	57.1 (21.1)	–	–
Oswestry Disability Index (0–100)	22.9 (11.1)	–	–
Number of pain sites			
Last 7 days	2.8 (1.9)	0.3 (0.7)	** < 0.001**
Last 12 months	5.5 (2.4)	1.4 (1.4)	** < 0.001**
Activity limiting in last 12 months	2.1 (1.8)	0.2 (0.5)	**< 0.001**
Pain medication usage n (%)*			
Yes	11 (52.4)	–	–
No	10 (47.6)	–	

Data is reported as mean and standard deviation unless otherwise specified.

*Participant 1: Panadol (Paracetamol) and Prozac (Anti-depressant)—last used 4 h prior to testing; Participant 2: Mobic (NSAID)—last used 72 h prior to testing; Participant 3: Voltaren (NSAID) and Panadol (Paracetamol)—last used 4 h prior to testing; Participant 4: Celebrex (NSAID) and Endone (Opioid)—last used 48 h prior to testing; Participant 5: Ibruprofen (NSAID)—last used 240 h prior to testing; Participant 6: Panadol (Paracetamol) and Celebrex (NSAID)—last used 21 days prior to testing; Participant 7: Panadol (Paracetamol)—last used 14 days prior to testing; Participant 8: Voltaren (NSAID) last used 36 h prior to testing; Participant 9: Panadol (Paracetamol)—time last used not reported; Participant 10: Voltaren (NSAID)—last used 96 h prior to testing; Participant 11: Ibruprofen (NSAID)/Paracetamol mixed tablet—last used 5 h prior to testing.

Significant values are in bold.

### Statistical and data-analytic results

*Step 1—Initial statistical tests:* Participants with CLBP differed from pain-free controls on multiple nervous system, spinal tissue and psychosocial factors (Supplementary Table 3). Of 54 variables in the primary analysis, 11 reached unadjusted statistically significant differences. These variables included: the number of pain sites over the last seven days and 12 months, central sensitisation inventory, satisfaction in social roles, depressive symptoms, maximal back extension strength, lumbar pressure-pain threshold, general self-efficacy, anxiety symptoms, cognitive function and average lumbar T2. Only the first three variables were statistically significant after adjustment for multiple comparisons. No variables reach the pre-determined cut-offs for multicollinearity (Supplementary Table 4; Supplementary Fig. 5).

*Step 2—Feature weighting between CLBP and controls:* The RF variable predictor for the primary analysis showed that the number of pain sites over the last seven days, over the last 12 months and central sensitisation inventory contributed the most to separating CLBP and pain-free controls (Supplementary Fig. 6). Given the importance of other variables in prior steps, we only removed pain sites over the prior seven days from subsequent analyses given its similarity to pain sites over the prior 12 months in feature weighting methods.

*Step 3—Feature ranking in CLBP only:* The factors with the most variance in CLBP participants were, in order of importance: cognitive function, depressive symptoms, anxiety symptoms, general self-efficacy, satisfaction in social roles, central sensitisation, pain site within the last 12 months, average lumbar pressure-pain thresholds, average lumbar T2 and maximal extension strength (Supplementary Fig. 7).

*Step 4—Cluster validity:* The allocation of participants with CLBP to clusters showed that adding more than four variables (in the order of importance determined in Step 3) led to decreases in clustering performance (Supplementary Table 5). Using four variables, CLBP participants were optimally classified into two clusters (Supplementary Table 5).

**Figure 2:**
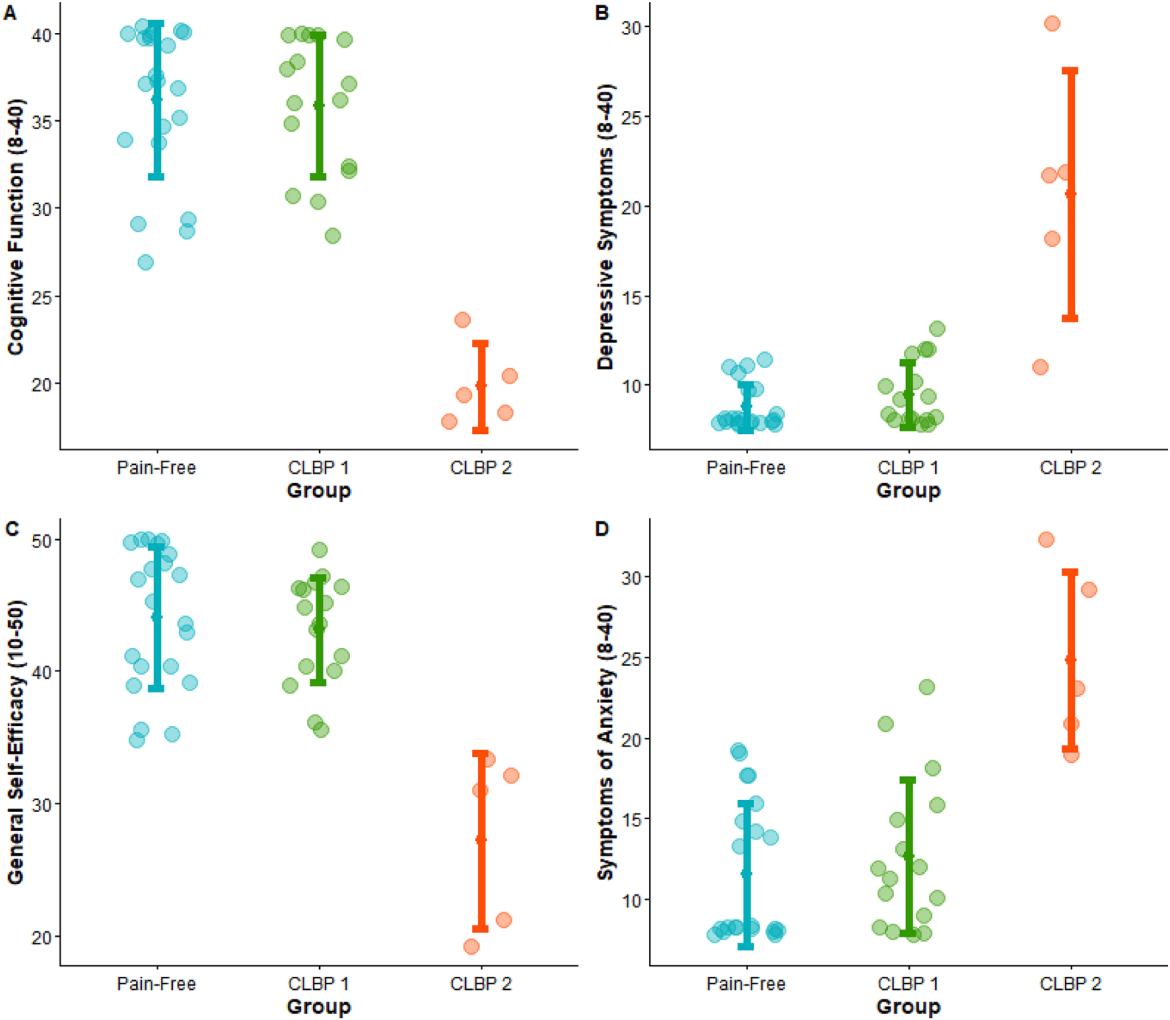
Plots of individual participant data points for cognitive function (**A**), depressive symptoms (**B**), general self-efficacy (**C**) and symptoms of anxiety (**D**) across pain-free (blue), CLBP sub-group #1 (green) and CLBP sub-group #2 (red) groups. Higher scores are better for cognitive functional and general self-efficacy. The error bars indicate the mean and standard deviation.

*Step 5—Clustering:* The two CLBP clusters (CLBP sub-group #1: normal psychosocial scores; CLBP sub-group #2: high psychosocial scores) were sub-grouped based on cognitive function, depressive symptoms, general self-efficacy and symptoms of anxiety through fuzzy c-means clustering (Fig. 2). The within-cluster distances on a normalised 0–1 scale were 0.28 for cluster one and 0.40 for cluster two, within a between-cluster distance of 0.53 (Supplementary Table 6). Post-hoc evaluation showed a Silhouette Index of 0.89, indicating good similarity of a data point, on average, to its cluster (Supplementary Fig. 8). The discrimination value of the clusters was − 2.3 (Supplementary Table 6).

*Step 6—Classification:* The classifiers fit to CLBP sub-groups showed that average error rates (95% confidence intervals) across the 30 runs of each classification model were 14.2 (8.6, 19.8)%, 4.2 (0.8, 7.6)%, 12.5 (7.4, 17.6)%, and 5.8 (2.0, 9.7)% for SVM, Naïve-Bayes, kNN, and RF classifiers, respectively (Supplementary Table 7). Adding pain-free controls to the data increased the error rates of classification models to 26.7 (22.2, 31.2)%, 24.2 (20.1, 28.2)%, 37.5 (32.5, 42.5)%, and 26.7 (20.0, 33.3)% for SVM, Naïve-Bayes, kNN, and RF classifiers, respectively (Supplementary Table 8).

*Step 7—Post-hoc statistical tests:* CLBP sub-group #1 differed from controls on the number of pain sites over 12 months (p < 0.001) and on the central sensitisation inventory (p = 0.005; Table 2). CLBP sub-group #2 differed from controls on pain sites over 12 months, central sensitisation, satisfaction in social roles, depressive symptoms, general self-efficacy, symptoms of anxiety and cognitive function compared to pain-free controls (all: p < 0.001; Table 2). CLBP sub-group #2 had higher levels of current (p = 0.041) and 1-week average pain-intensity (p = 0.013) and disability (p = 0.015) compared to sub-group #1 (Table 2).

**Table 2:** Descriptive statistics of pain-free and derived CLBP sub-groups from the primary analysis.

	Pain-free (n = 21)	CLBP sub-group #1 (n = 16)	p-value	CLBP sub-group #2 (n = 5)	p-value	p-value between CLBP groups
Ages (years)	35.6 (11.1)	34.9 (11.2)	0.981	37.0 (11.5)	0.967	0.932
Sex (male/female—n)	10/11	8/8	–	2/3	–	0.927**
Height (cm)	172.9 (9.1)	173.8 (10.7)	0.963	174.7 (9.7)	0.925	0.979
Body mass index (kg/cm^2^)	25.3 (6.1)	27.5 (5.3)	0.473	26.3 (4.5)	0.930	0.912
Pain intensity (0–100)						
Current	–	27.8 (19.2)	–	45.0 (17.5)	–	**0.041**
Last-week average	–	34.1 (16.8)	–	52.0 (14.4)	–	**0.013**
Last-week worst	–	53.8 (20.7)	–	67.8 (20.6)	–	0.175
Oswestry Disability Index (0–100)	–	20.3 (9.3)	–	31.2 (13.2)	–	**0.015**
Number of pain sites over the last 12 months	1.4 (1.4)	5.2 (2.5)	** < 0.001**	6.6 (1.9)	** < 0.001**	0.345
Central Sensitisation Inventory (0–100)	17.1 (10.3)	32.3 (11.8)	**0.005**	51.2 (11.1)	** < 0.001**	**0.005**
Satisfaction in social roles (8–40)*	38.0 (3.8)	34.8 (7.7)	0.232	24.8 (7.4)	** < 0.001**	**0.007**
Depressive symptoms (8–40)	8.8 (1.3)	9.4 (1.8)	0.734	20.6 (6.9)	** < 0.001**	** < 0.001**
Maximal Extension Strength (kg)	68.7 (16.2)	53.7 (21.8)	0.170	49.2 (17.9)	0.104	0.679
Average lumbar pressure-pain thresholds (kg/cm^2^)	9.1 (2.2)	7.6 (2.7)	0.186	6.7 (2.4)	0.131	0.729
General self-efficacy (10–50)*	44.1 (5.4)	43.1 (4.0)	0.831	27.2 (6.6)	** < 0.001**	** < 0.001**
Symptoms of anxiety (8–40)	11.5 (4.4)	12.6 (4.8)	0.759	24.8 (5.5)	** < 0.001**	** < 0.001**
Cognitive function (8–40)	36.2 (4.4)	35.8 (4.1)	0.958	19.8 (2.5)	** < 0.001**	** < 0.001**
Average lumbar T2-time (ms)	103.4 (13.5)	95.1 (9.9)	0.124	96.9 (15.7)	0.551	0.958

Data are reported as mean and standard deviation unless otherwise specified. All p-values are Tukey HSD adjusted through between-group ANOVAs.

*Higher values are better.

**Results of chi-square test between all groups.

Significant values are in bold.

**Figure 3:**
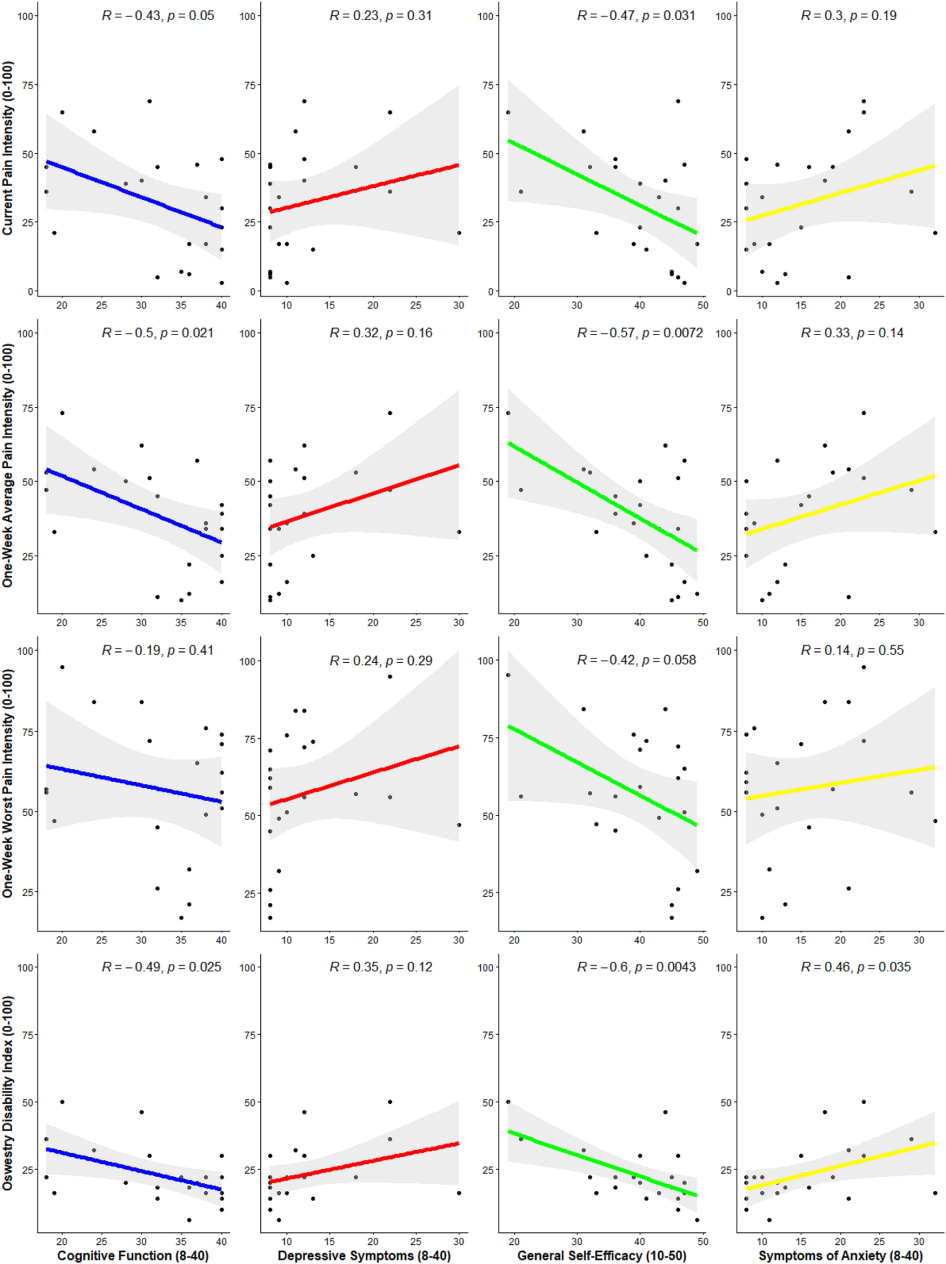
Scatter plots indicated the Pearson’s correlation coefficient and 95% confidence interval between factors deriving the CLBP sub-groups in the primary analysis and pain intensity and disability.

From the main variables used to derive CLBP sub-groups, there were significant correlations between cognitive function and 1-week average pain intensity (r = − 0.50, p = 0.021) and disability (r = − 0.49, p = 0.025). There were also significant correlations between general self-efficacy and current pain intensity (r = − 0.47, p = 0.031), 1-week average pain intensity (r = − 0.57, 0.007) and disability (r = − 0.60, p = 0.004). Lastly, there was a significant correlation between symptoms of anxiety and disability (r = 0.46, p = 0.035; Fig. 3).

### Secondary analyses using additional variables

*Step 8—Secondary analyses:* Results of the secondary analyses using the additional variables pre-defined in Table 3 are reported in Supplementary Tables 9–14 and Supplementary Figs. 9–13. After the assessment of t-tests, multicollinearity, feature weighting and Laplacian scores, cognitive function, depressive symptoms, general self-efficacy, maximum facet joint grading, anxiety symptoms, satisfaction in social roles, central sensitisation inventory, maximal extension strength, L2 quadratus lumborum fat fraction, number of pain sites over the last 12 months, average back pressure-pain thresholds, L5S1 T2, L4L5 T2 and maximal Pfirrmann grade, were, in order of importance, used in data-analytic steps. Overall, two clusters using three variables of cognitive function, depressive symptoms, and general self-efficacy were derived. Results of classification accuracy were similar to the primary analyses.

**Table 3:** Factors included across biopsychosocial domains in primary and secondary analyses.

Nervous system	Spinal tissues	Psychosocial
Factors used in primary data-analytic methods		
*Grey matter volumes (mm* ^*3*^ *)* Medial frontal cortex, amygdala, thalamus, insula, caudate, putamen, anterior cingulate cortex, hippocampus, precentral gyrus (primary motor cortex), supplementary motor cortex, post-central gyrus (primary somatosensory cortex) and parietal operculum (secondary somatosensory cortex) pooled across left and right sides *Functional connectivity (z)* Medial prefrontal cortex to nucleus accumbens Posterior cingulate cortex to angular gyrus *Pressure-pain thresholds (kg/cm* ^*2*^ *)* Forearms, lumbar spine, and posterior calves pooled across left and right sides *Temporal summation (change in vNRS; 0–10)* Forearms, lumbar spine, and posterior calves pooled across left and right sides *Exercise induced hypoalgesia (change in pressure pain thresholds; kg/cm* ^*2*^ *)* Forearms, lumbar spine, and posterior calves pooled across left and right sides *Other* Central sensitisation inventory (0–100)	*Intervertebral disc height (mm)* Average height across all lumbar levels (from middle three slices surrounding the spinous process) *Intervertebral disc volume (cm* ^*3*^ *)* Average disc volume across all lumbar levels *Intervertebral disc* T2* (ms)* Average T2 across all lumbar levels (from central three slices at the spinous process) *Vertebrae fat fraction (%)* Average fat fraction across all lumbar levels (from three highest contiguous slices) *Paraspinal muscle volume (cm* ^*3*^ *)* Average volume of the multifidus, erector spinae, psoas major and quadratus lumborum across the lumbar levels pooled across sides *Paraspinal muscle fat fraction (%)* Average fat fraction of the multifidus, erector spinae, psoas major and quadratus lumborum across the lumbar levels (from middle three slices at each level) pooled across sides *Radiographic grading* Average Pfirrmann grading across all lumbar levels (1–5) Average facet joint grading across lumbar levels (0–3) Average pars grading across all lumbar levels (0–4) Average disc bulge grade across all lumbar levels (0–3) *Trunk muscle endurance (s)* Maximal trunk flexion and extension endurance *Trunk muscle strength (kg)* Maximal trunk extension strength	*PROMIS questionnaires* Anxiety (8–40) Depression (8–40) Cognitive function (8–40; higher scores are better) General self-efficacy (10–50; higher scores are better) Satisfaction in social roles and activities (8–40; higher scores are better) Social isolation (8–40) Emotional support (8–40; higher scores are better) Instrumental support (8–40; higher scores are better) *Other factors that do not fit the three domains* Nordic musculoskeletal questionnaire – average number of pain sites over the last 7 days and 12 months Body mass index (kg/m^2^)
Factors used in secondary data-analytic methods^a^		
*Grey matter volumes (mm* ^*3*^ *)* Medial frontal cortex, amygdala, thalamus, insula, caudate, putamen, anterior cingulate cortex, hippocampus, precentral gyrus (primary motor cortex), supplementary motor cortex, post-central gyrus (primary somatosensory cortex) and parietal operculum (secondary somatosensory cortex) side specific *Functional connectivity (z)* Medial prefrontal cortex to nucleus accumbens Posterior cingulate cortex to angular gyrus *Pressure-pain thresholds (kg/cm* ^*2*^ *)* Forearms, lumbar spine, and posterior calves side specific *Temporal summation (change in verbal numeric rating scale; 0–10)* Forearms, lumbar spine, and posterior calves side specific *Exercise induced hypoalgesia (change is pressure pain thresholds; kg/cm* ^*2*^ *)* Forearms, lumbar spine, and posterior calves side specific *Other* Central sensitisation inventory (0–100)	*Intervertebral disc height (mm)* Lumbar level specific disc height (from middle three slices surrounding the spinous process) *Intervertebral disc volume (cm* ^*3*^ *)* Lumbar level specific disc volume *Intervertebral disc* T2* (ms)* Lumbar level specific T2 across (from middle three slices surrounding the spinous process) Lumbar level specific nucleus only T2 across (from middle three slices surrounding the spinous process) Lumbar level specific average T2 across (across whole disc) *Vertebrae fat fraction (%)* Lumbar level specific fat fraction (from three highest contiguous slices) *Paraspinal muscle area (mm* ^*2*^ *)* Lumbar level specific muscle size (from middle three slices at each level) *Paraspinal muscle fat fraction (%)* Lumbar level specific fat fraction (from middle three slices at each level) *Radiographic grading* Highest Pfirrmann grading across all lumbar levels (1–5) Highest facet joint grading across lumbar levels (0–3) Highest pars grading across all lumbar levels (0–4) Highest disc bulge grade across all lumbar levels (0–3) *Trunk muscle endurance (s)* Maximal trunk flexion and extension endurance *Trunk muscle strength (kg)* Maximal trunk extension strength	*PROMIS questionnaires:* Anxiety (8–40) Depression (8–40) Social isolation (8–40) Cognitive function (8–40; higher scores are better) General self-efficacy (10–50; higher scores are better) Satisfaction in social roles and activities (8–40; higher scores are better) Emotional support (8–40; higher scores are better) Instrumental support (8–40; higher scores are better) *Other Factors that do not fit the three domains:* Nordic musculoskeletal questionnaire – average number of pain sites over the last 7 days and 12 months Body mass index (kg/m^2^)

Data used in primary and secondary analyses was pre-registered on the Open Science Framework (https://osf.io/b4edg/).

*In the instance where left- and right-hand sides data are highly correlated (r > 0.80), we used the pooled value in our secondary analysis; using the highest/lowest level of spinal tissue was used to reflect the most affected level.

### Secondary analyses in sub-domains

*Step 9—Sub-domain analyses:* Deriving sub-groups in each sub-domain was used to overcome differences in variance between factors as a sensitivity analyses^4^. Variables which passed feature weighting in the primary results were used in the relevant sub-domain analyses.

*Psychosocial:* Given psychosocial factors derived sub-groups in both the primary and secondary analyses, we did not complete further clustering within this domain.

*Spinal tissue:* Results of the spinal tissue only data-analytic results are in Supplementary Tables 15–19 and Supplementary Figs. 14–16. Two clusters were optimal for deriving sub-groups based on maximal lumbar extension strength and average lumbar T2. Classification accuracy was like the primary analyses. The two derived sub-groups consisted of one with low maximal lumbar extension strength and T2, and one with normal values compared to pain-free controls (Supplementary Table 19; Supplementary Fig. 15). There were no statistically significant correlations between maximal lumbar extension strength and average lumbar T2 and pain intensity and disability (Supplementary Fig. 16).

*Nervous system:* Results of the nervous system only results are in Supplementary Tables 20–24 and Supplementary Figs. 17–19. Five clusters based on central sensitisation and average lumbar PPTs were optimal for deriving CLBP sub-groups. Classification error increased across the models (Supplementary Tables 22–23). The five sub-groups, which were compared to pain-free controls, consisted of: (1) low lumbar PPTs, (2) no nervous system contribution, (3) high central sensitisation and low lumbar PPTs, (4) moderate central sensitisation and (5) high central sensitisation (Supplementary Table 19; Supplementary Fig. 18). There was a significant correlation between central sensitisation and 1-week average pain intensity (r = 0.50, p = 0.022) and disability (r = 0.55, p = 0.010; Supplementary Fig. 19).

Overall, eight participants (38%) were classified as having nervous system contributions only, four (19%) as spinal tissue only, four (19%) as nervous system and spinal tissue, two (9.5%) as having psychosocial and nervous system and three (14.3%) as having spinal tissue, psychosocial and nervous system contributions (Table 4).

**Table 4:** Classification in each of the psychosocial, spinal tissue and nervous system from clusters derived within each of the sub-domains.

Participant	Psychosocial classification^a^	Spinal tissue classification	Nervous system classification	Overall classification	Domains contributing	Average 1-week pain intensity (0–100)	Oswestry Disability Index (0–100)
1	Normal psychosocial scores	Normal lumbar extension strength and T2	Low lumbar pressure-pain threshold only	Low lumbar pressure-pain threshold only	Nervous system	34	10
2	High psychosocial scores	Low lumbar extension strength and T2	High central sensitisation only	High psychosocial scores; Low lumbar extension strength and T2; High central sensitisation	Psychosocial; spinal tissue; nervous system	47	36
3	Normal psychosocial scores	Low lumbar extension strength and T2	Normal central sensitisation and lumbar pressure-pain threshold	Low lumbar extension strength and T2	Spinal tissue	10	22
4	High psychosocial scoress	Normal lumbar extension strength and T2	High central sensitisation and low lumbar pressure-pain threshold	High psychosocial scores; High central sensitisation and low lumbar pressure-pain threshold	Psychosocial; nervous system	33	16
5	High psychosocial scores	Low lumbar extension strength and T2	High central sensitisation and low lumbar pressure-pain threshold	High psychosocial scores; Low lumbar extension strength and T2; High central sensitisation and low lumbar pressure-pain threshold	Psychosocial; spinal tissue; nervous system	73	50
6	Normal psychosocial scores	Normal lumbar extension strength and T2	High central sensitisation only	High central sensitisation only	Nervous system	34	16
7	Normal psychosocial scores	Low lumbar extension strength and T2	High central sensitisation and low lumbar pressure-pain threshold	Low lumbar extension strength and T2; High central sensitisation and low lumbar pressure-pain threshold	Spinal tissue; nervous system	62	46
8	Normal psychosocial scores	Low lumbar extension strength and T2	Normal central sensitisation and lumbar pressure-pain threshold	Low lumbar extension strength and T2	Spinal tissue	16	16
9	Normal psychosocial scores	Low lumbar extension strength and T2	Low lumbar pressure-pain threshold only	Low lumbar extension strength and T2; Low lumbar pressure-pain threshold only	Spinal tissue; nervous system	39	22
10	High psychosocial scores	Low lumbar extension strength and T2	High central sensitisation only	High psychosocial scores; Low lumbar extension strength and T2; High central sensitisation	Psychosocial; spinal tissue; nervous system	54	32
11	Normal psychosocial scores	Low lumbar extension strength and T2	Normal central sensitisation and lumbar pressure-pain threshold	Low lumbar extension strength and T2	Spinal tissue	25	14
12	Normal psychosocial scores	Low lumbar extension strength and T2	High central sensitisation only	Low lumbar extension strength and T2; High central sensitisation	Spinal tissue; nervous system	51	30
13	Normal psychosocial scores	Normal lumbar extension strength and T2	High central sensitisation only	High central sensitisation only	Nervous system	57	20
14	Normal psychosocial scores	Normal lumbar extension strength and T2	High central sensitisation only	High central sensitisation only	Nervous system	12	6
15	Normal psychosocial scores	Low lumbar extension strength and T2	Normal central sensitisation and lumbar pressure-pain threshold	Low lumbar extension strength and T2	Spinal tissue	42	30
16	Normal psychosocial scores	Normal lumbar extension strength and T2	High central sensitisation only	High central sensitisation	Nervous system	11	14
17	High psychosocial scores	Normal lumbar extension strength and T2	High central sensitisation only	High psychosocial scores; High central sensitisation	Psychosocial; nervous system	53	22
18	Normal psychosocial scores	Normal lumbar extension strength and T2	Low lumbar pressure-pain threshold only	Low lumbar pressure-pain threshold	Nervous system	36	22
19	Normal psychosocial scores	Low lumbar extension strength and T2	High central sensitisation and low lumbar pressure-pain threshold	Low lumbar extension strength and T2; High central sensitisation and low lumbar pressure-pain threshold	Spinal tissue; Nervous system	45	18
20	Normal psychosocial scores	Normal lumbar extension strength and T2	High central sensitisation only	High central sensitisation only	Nervous system	22	18
21	Normal psychosocial scores	Normal lumbar extension strength and T2	High central sensitisation only	High central sensitisation only	Nervous system	50	20

^a^High psychosocial symptoms include cognitive function, depressive symptoms, general self-efficacy, and symptoms of anxiety.

## Discussion

This pilot study classified CLBP participants into sub-groups using machine learning. In our sample, two sub-groups of participants with CLBP were derived primarily based on psychosocial factors of cognitive function, depressive symptoms, general self-efficacy and symptoms of anxiety. Classification accuracy was over 80% when only CLBP sub-groups were considered and 62% when pain-free controls data were added. Secondary sub-domain analyses derived two additional sub-groups based on spinal tissue factors of maximal lumbar extension strength and average lumbar intervertebral disc T2, and five nervous system sub-groups based on central sensitisation and lumbar PPTs.

The results of our study are congruent with our previous retrospective analyses of the UKBioBank (n = 19,083) which accurately classified chronic back pain patients into sub-groups based on depressive symptoms and loneliness/social isolation^13^. These findings suggest that psychosocial factors have more variance than spinal tissue and nervous system factors in chronic low back pain and dictate clustering. Feature weighting and validity methods assessed the data based on distances within- and between-groups^14,15^. After scales were normalised, psychosocial factors demonstrated greater variance across the scale and came out as the most important discriminating factors. Notably, only 5/21 (24%) of participants were classified into the sub-group with higher severity psychosocial scores. Therefore, future studies should evaluate classifications across similar domains of outcomes.

Our secondary sub-domain analyses identified spinal tissue sub-groups based on maximal lumbar extension strength and average lumbar T2, and nervous system sub-groups based on central sensitisation and average lumbar PPTs. These results demonstrate that sub-groups derived from different sub-domains overcame variance differences in multidimensional subjective and objective factors^14,15^. For example, Table 4 demonstrated that there was a broader range of potential CLBP profiles when considering the label of each sub-domain. Furthermore, correlation analyses showed that no factors were highly correlated across sub-domains, which may highlight the distinct mechanisms of each variable to the pain experience^6^. Our results indicate that future research should derive sub-groups and attempt classification on each sub-domain.

An important novelty of our study was the measure of spinal tissue factors using MRI. Our systematic review showed that only four studies had previously assessed spinal tissues in conjunction with psychosocial factors^4^. Whilst poor spinal tissue health does not always result in pain^16^, the factors we measured have previously been associated with low back pain^10^. Given the potential ongoing nociception contributing to CLBP^17^, understanding the interaction of spinal tissues with psychosocial and nervous system factors warrants attention. Our primary results showed that 11/21 (52%) of CLBP participants had significantly lower maximal lumbar extension strength and average lumbar disc T2-time compared to pain-free controls. Of these participants, four were classified as spinal tissue only, four as nervous system and spinal tissue, and three as having spinal tissue, psychosocial and nervous system contributions. CLBP participants who had contributions from all domains had higher 1-week average pain intensity and disability compared to those classified as spinal tissue only (Table 4). A combination of spinal tissue, psychosocial and nervous system domains may contribute to higher levels of pain intensity and disability, however, these findings need to be confirmed in larger samples.

The secondary analyses showed that specific lumbar level factors may be the most important contributor in the spinal tissue domain. For example, maximum facet joint grading, L2 quadratus lumborum fat fraction, L5-S1 T2, L4-L5 T2 and maximal Pfirrmann grade were important contributors to pain following feature weighting. Exploring this further in larger samples may assist in identifying individuals with lumbar level specific CLBP. For example, our correlation analyses showed a moderate (r = 0.30) association between L5S1 intervertebral disc T2 and L4L5 intervertebral disc T2, meaning that different lumbar levels may independently contribute to CLBP. Future research should examine the interaction of multidimensional classification on pain intensity and disability and consider lumbar level specific factors on overall classification methods.

Changes to the structure and function of the brain have been observed in individuals with CLBP^7^. Prior research (n = 11,106) reported differences in grey matter volumes in the primary motor and somatosensory cortices, caudate and amygdala exist between chronic back pain (localised or widespread) and pain-free controls^9^. These findings were not replicated here. The effect sizes of differences in grey matter volumes was noted to be very small (Cohens d: < 0.2)^9^, and given our limited sample size, may have not been powered enough to detect differences in grey matter volumes between groups. For functional connectivity, we used known seeds in the DMN^8,18^, however, did not see any differences. The sample sizes across these studies^8,18^ and reviews in the area^7,19^, are small (n < 100) and may explain the variability in results. Meta-analysis could be used to overcome this limitation in neuroimaging, however individual studies normally only report on specific brain regions^7,19^. Therefore, future research on brain structure should consider samples sizes and standard connectivity reporting to determine the most appropriate brain hubs for CLBP conditions.

The clinical relevance of this research is that multidimensional classification of CLBP should occur before initiating treatment for CLBP^6^. Not all individuals will have important findings across all domains (Table 4). Patients classified with mainly spinal tissue contributions to pain should be treated differently than patients where neurological, psychological or social factors predominate. Our results suggest that psychosocial factors, were the most useful to classify CLBP patients. Until more robust data-driven classification are developed, clinically implementable questionnaires such as the STarTBack Tool (physical and psychosocial)^20^, central sensitisation inventory (part A; nervous system)^20^ and Orebro Musculoskeletal Questionnaire (physical and psychosocial)^21^ could be used with objective factors of maximal extension strength (spinal tissue) and pressure-pain thresholds (nervous system), and other known contributors to CLBP, to determine the potential contribution of different domains to the condition in clinical practice. These results support multidimensional considerations in clinical practice to move to more prominent patient-centred care.

Strengths of the current study include that it is the first study to consider a broad range of spinal tissue, psychosocial and nervous system factors in the same participants with CLBP. We also matched participants on age, sex, height and body mass index (where possible). Moreover, we completed secondary and sub-domain analyses to overcome the variance in different factors and derive overall classification across all the sub-domains. Finally, we correlated the factors deriving sub-groups to pain intensity and disability to improve the real-world applicability.

In terms of limitations, first is the small sample of CLBP (n = 21) and pain-free (n = 21) participants that allowed running the data-driven model but lacked generalisability and statistical power for secondary analyses. Future research with larger samples should attempt classification within each sub-domain to best separate individuals with CLBP from pain-free controls. Second, while our list of multidimensional factors was exhaustive, more outcomes could have been added to the model. Third, we selected factors associated with CLBP however, such a cross-sectional study cannot infer causality. Finally, given the pilot nature of the study and small sample size, we could not complete important steps of evaluation for clinical prediction models including external validation, calibration, stability assessment and net-benefit analyses^22–24^. Therefore, these should be conducted with larger samples to ensure a robust classification system.

In conclusion, this pilot study was the first to consider a wide range of spinal tissue, nervous system and psychosocial factors to improve the data-driven classification of non-specific CLBP. The findings attest to the feasibility of the approach and support developing data-driven classification of non-specific CLBP. In our study, two CLBP sub-groups were derived on psychosocial factors of cognitive function, depressive symptoms, general self-efficacy and symptoms of anxiety. The classification accuracy was above 80% for CLBP participants. Secondary analyses suggested deriving sub-domain classifications. Future research should optimise the methods used in this study with larger samples to improve multidimensional data-driven classification of CLBP, a prerequisite to the targeted, logical management of individuals with CLBP.

## Methods

This was a pilot cross-sectional study of 21 individuals with non-specific CLBP and 21 age-, sex-, and self-report height-matched pain-free controls between the ages of 18–55 years. Ethical approval was granted by the Deakin University Human Research Ethics Committee (project ID: 2020-124) and conducted in line with the Declaration of Helsinki. All participants provided written and informed consent prior to study participation. We report this study in concordance with Strengthening the Reporting of Observational Studies in Epidemiology (STROBE) guidelines^25^. The code and anonymised data for this study are available on the Open Science Framework (https://osf.io/b4edg/).

### Recruitment

Community-dwelling individuals were recruited from the greater metropolitan region of Melbourne (Victoria, Australia). Social media advertising and print-based flyers were used to assist with recruitment. Participants from prior studies^26–28^ who gave consent to be contacted for future studies were also contacted. Potential participants registered interest through a study specific website and were screened via telephone against a priori inclusion and exclusion criteria.

### Inclusion criteria and exclusion criteria

Participants were recruited and stratified according to age groups (n = 5:5:5:6 in 18–25 yr, 26–35 yr, 36–45 yr and 46–55 yr). Inclusion criteria for the non-specific CLBP group were a self-reported episode of pain between the T12 vertebrae and gluteal fold, with or without leg pain, that had lasted for more than 12 weeks^29,30^. CLBP participants were also required to have a pain intensity of at least 3/10, on average of the prior week, on the verbal numeric rating scale at the time of telephone screening^31^. Individuals with co-morbid non-specific CLBP and diagnosed depression and/or anxiety were included.

Exclusion criteria for both groups were: (1) history of spinal surgery, (2) history of spine trauma (e.g. fracture), (3) cauda equina symptoms, (4) known structural scoliosis, (5) diagnosed radiculopathies, (6) inflammatory spondyloarthropathies, (7) non-musculoskeletal causes of LBP (e.g. infection, visceral pain), (8) inability to communicate in English, (9) pregnancy, current lactation or < 1 year postnatal, (10) current or prior elite athletes (i.e. member of Australian Institute of Sport, State Institutes or Academies of Sport, the national squad of any sport, or playing in a professional sporting league)^32^, and (11) any absolute contraindications for magnetic resonance imaging (MRI) or exercise testing^33^. We also excluded individuals with diagnosed neurological conditions (e.g. stroke and multiple sclerosis), prior major head trauma or brain surgery and those with major psychiatric disorders (e.g. schizophrenia and bipolar disorders).

The following exclusion criteria also applied for the pain-free group: (1) current spinal (neck, upper back, or low back) pain, (2) back pain lasting for more than 24 h within the last year (except for muscle soreness related to physical activity), (3) had previously, at any point, missed days from work due to back pain and (4) had previously, at any time, visited a health professional for medical treatment of back pain (e.g. physiotherapist and general practitioner).

### Matching criteria

Pain-free controls were matched to CLBP participants by sex (male or female), age brackets (18–25 yr, 26–35 yr, 36–45 yr and 46–55 yr) and height (± 5cm). Where possible, it was also attempted to match participants within a body mass index of ± 5kg/m^2^. Due to COVID-19 restrictions in Melbourne (Victoria, Australia), self-report height and weight collected during telephone screening were used for matching. Where participants matched on sex, age, and self-report height, but not body mass index, we included the participant with the closest available body mass index.

### Data collection

Data collection consisted of two testing sessions. The first testing session was conducted at Imaging@OlympicPark (Melbourne, Victoria, Australia) where participants underwent spinal tissue MRI and physical testing. The second testing session was conducted at Monash Biomedical Imaging (Clayton, Victoria, Australia) where participants underwent brain MRI.

Variables were collected under the following domains (Table 3): (1) nervous system: grey matter volumes, resting-state functional connectivity, pressure-pain thresholds, temporal summation, exercise-induced hypoalgesia and central sensitisation inventory; (2) spinal tissue: intervertebral disc height, volume and T2-time, vertebral body fat fraction and paraspinal muscle volume, size and fat fraction, lumbar radiographic grading, trunk muscle strength and endurance; and (3) psychosocial: anxiety, depression, cognitive function, general self-efficacy, satisfaction in social roles and activities, social isolation, and social and instrumental support. Pain intensity and disability were collected to help characterise derived sub-groups. All questionnaires and physical variables were collected and recorded using an online database (Qualtrics, Seattle, United States of America). Pre-specified variables used in our primary and secondary analyses are reported on the Open Science Framework (https://osf.io/b4edg/).

### Nervous system

#### Grey matter volumes

Brain imaging was completed using a SIEMENS Skyra 3.0-T MRI (Siemens Healthineers, Erlangen, Germany). T1-weighted MPRAGE anatomical images (frames: 192, repetition time: 1900.0 ms, echo time: 2.16 ms, flip angle: 9°, field-of-view: 288 × 288 pixels, bandwidth: 230 Hz) were used to estimate cortical and subcortical grey matter volumes using FSL packages (version 6.0; FMRIB software library, Oxford, England; http://fsl.fmrib.ox.ac.uk/fsl). FSL was run through Ubuntu 18.04 Bionic (https://releases.ubuntu.com/18.04.5/) on Windows 10 using the Xming graphical user interface (https://sourceforge.net/projects/xming/). Images were firstly converted from DICOM to NIfTI format using dcm2nii (https://www.nitrc.org/projects/dcm2nii/). Following this, images underwent processing through FSL using the ‘fslanat’ (https://fsl.fmrib.ox.ac.uk/fsl/fslwiki/fsl_anat), which implements the following steps: (a) change images to MNI orientation (fslreorient2std), (b) crop the images to remove excessive non-brain tissue (robustfov), (c) bias field inhomogeneity correction (FAST), (d) linear (FLIRT) and non-linear (FNIRT) registration of images to standard space, (e) brain extraction (FNIRT based), (f) tissue type segmentation into partial volume estimation of cerebrospinal fluid, grey matter and white matter (FAST) and (g) segmentation of subcortical structures (FIRST). We used the partial grey matter volume estimate output by FAST to calculate regional volumes in regions of interest using Harvard–Oxford cortical and sub-cortical atlases and Cerebellum atlas available in FSL. Given atlases are in MNI152 space, we used the estimated warp field from non-linear registration and inversely applied (invwarp followed by applywarp) this to generate atlases in the native space of the participant. Masks of regions of interest were then generated (fslmaths) and volumes in mm^3^ estimated (fslstats). For our study, we used regional grey matter volumes commonly linked to pain processing, which include the medial frontal cortex, amygdala, thalamus, insula, caudate, putamen, anterior cingulate cortex, hippocampus, precentral gyrus (primary motor cortex), supplementary motor cortex, post-central gyrus (primary somatosensory cortex) and parietal operculum (secondary somatosensory cortex)^9,34^.

#### Resting-state functional connectivity

Resting-state functional MRI (rsfMRI) simultaneous multi-slice sequences (frames: 490, repetition time: 736.0 ms, echo time: 39.0 ms, flip angle: 52°, field-of-view: 704 × 704 pixels, bandwidth: 2030 Hz) were collected on a SIEMENS Skyra 3.0-T magnetic resonance imaging (Siemens Healthineers, Erlangen, Germany). Following the completion of the rsfMRI sequences, using the same scanner, fieldmap magnitude images (frames: 128, repetition time: 674.0 ms, echo time one: 4.92 ms, echo time two: 7.38 ms, flip angle: 60°, field-of-view: 96 × 96 pixels, bandwidth: 330 Hz) were collected to use in distortion correction. Phase difference images were calculated within the scanning protocol.

rsfMRI image pre-processing, denoising and first-level analyses (collection of single subject functional connectivity) were completed using the Conn toolbox in MATLAB 2020a (MathWorks, Sherbon, United States of America). The steps of pre-processing included removal of the first five volumes, skull stripping, distortion correction using fieldmap magnitude and phase differences images, slice timing correction (SIEMENS interleaved), motion correction (head motion threshold set at 3 mm), co-registration of structural and functional images, segmentation of structural images into white and grey matter and cerebral spinal fluid and registration of images to MNI space. Functional images were also smoothed at a 5 mm FWHM Gaussian kernel. The images were denoised (physiological noise removal) using a 0.008–0.09Hz band-pass filter using the CompCor method^35^.

The default mode network (DMN) has been implicated as an important resting-state brain network for CLBP, with primary functional connectivity hubs including the posterior cingulate cortex (PCC) and the medial prefrontal cortex (mPFC)^7^. For the first-level analyses, prior work^8^ was followed and 10mm spheres were created based on a prior meta-analysis^36^ to use as seeds in the PCC (x, y, z = − 8, − 56, 39) and mPFC (x, y, z = 4, 42, 3). These spheres were created using the Marsbar toolbox^37^. Given thousands of brain connections exist, to limit the number of variables, the connectivity of the posterior cingulate cortex to the angular gyrus (AG; x, y, z = − 52, − 66, 36)^8^ and the medial prefrontal cortex to the nucleus accumbens (NAc; x, y, z = 10, 12, − 8)^18^ were extracted due to their importance in prior research (Supplementary Fig. 1). Correlation coefficients (Fisher-transformed) across the time series between the PCC-AG and mPFC-NAc were used in subsequent analyses.

#### Pressure-pain thresholds

Pressure-pain thresholds (PPT) were assessed bilaterally at muscle bellies of the forearms, calves and lumbar paraspinals using established and reliable protocols^38,39^. The specific anatomical locations were: (1) forearms: with the participant prone on a plinth, hands were pronated and placed underneath the forehead and pressure was applicated 3 cm posteriorly and distally to the lateral epicondyle of the humerus; (2) calves: with the participant lying prone and the feet in a neutral position, slightly hanging off the plinth, pressure was applied to a site around the proximal third of the tibia to capture the muscle belly of the gastrocnemius; and (3) lumbar paraspinals: with the participant in prone, pressure was applied at approximately the L4 level by palpating the iliac crests and applying pressure four centimetres from the midline. Manual pressure was applied using a digital algometer (Commander Echo, J Tech Medical Industries, Salt Lake City, United States of America) at a rate of approximately 1 kg/s until the participant said ‘pain’ or ‘stop’ at the point pressure turned to pain. For participant safety, the algometer was set to achieve a maximum of 11.3 kg/cm^2^. The tests were conducted at the anatomical locations (L, R, L, R) in a randomised order with a minimum of 20 s rest between trials at the same location. The average of the two tests at each location in kg/cm^2^ was used in analyses.

#### Temporal summation of pain

Temporal summation of pain was assessed by applying 10 consecutive pressure stimuli using a digital algometer (Commander Echo, J Tech Medical Industries, Salt Lake City, United States of America) at the same locations of PPTs^40,41^. Pressure was increased at a rate of approximately 2 kg/s and once the previously determined average PPT of the same anatomical location was reached, the stimuli were held for one second. Each pulse was separated by one second. Participants were asked to rate their perceived pain intensity of the first, fifth and tenth pulse using a verbal numeric rating scale of zero (no pain) to 10 (most severe pain imaginable). The temporal summation of pain score at each anatomical location used for analyses was determined by subtracting the first pulse from the tenth pulse.

#### Exercise-induced hypoalgesia

To determine exercise-induced hypoalgesia, PPTs were reassessed immediately following an isometric wall squat maintained for three-minutes or until volitional fatigue^38,39,42^. The difference in PPT at each anatomical location before and immediately after the isometric wall squat was used to determine the magnitude of exercise-induced hypoalgesia and used for analyses. From this, positive values indicate an increase and negative values denote a decrease, in pressure-pain thresholds in kg/cm^2^.

#### Central Sensitisation Inventory

The Central Sensitisation Inventory is a self-report questionnaire to assess the presence of central sensitisation used in this study as a proxy measure of central nervous system hypersensitivity which is known to be present in some individuals with back pain^43^. The questionnaire has been established for reliability and validity^44^.

### Spinal tissues

#### Scanning protocols and region-of-interest tracing

All spinal imaging was conducted using an MRI scanner (Ingenia 3.0 T, Philips Healthcare, Macquarie Park, Australia). To avoid the impact of diurnal variation^45^ and physical activity^46^ on the spine, all scanning was performed at least four hours after the participant waking and participants were instructed not to complete any strenuous physical activity or sport on the day of scanning. Furthermore, participants were required to sit quietly for a minimum of 20 min prior to scanner entry. The following scanning protocols were performed:

1. Sagittal spin-echo multi-echo sequences with spinal coils was used to collect eight echo time (15.75, 36.75, 57.75, 78.75, 99.75, 120.75, 141.75 and 162.75 ms) across 12 sagittal slices (slice thickness: 3.5 mm, inter-slice distance: 1.0 mm, repetition time: 2000 ms, field-of-view: 704 × 704 pixels, bandwidth: 142.0 Hz) to encompass the entire lumbar spine from left to right. Spin-echo sequences were used for quantifying the intervertebral discs in each subject.
2. A 65-slice true-axial Dixon sequence (slice thickness: 3.5 mm, inter-slice distance: 0 mm, repetition time: 3.64 ms, echo times: 1.19/2.37 ms, field-of-view: 250APx300RLmm interpolated to 432 × 432 pixels, bandwidth: 1381.0 Hz) was used to encompass images from the sacrum up to and including T12 vertebra. True-axial Dixon sequences were used for measuring the vertebral bodies and paraspinal muscles in each subject.

To ensure blinding of case–control studies, images were assigned a random number (obtained from http://www.random.org) prior to region-of-interest tracing by S.D.T. ImageJ (v1.51j8, https://imagej.nih.gov/ij/) was used to trace the intervertebral discs, vertebral bodies and paraspinal muscles on relevant images. A custom ImageJ plugin was used to quantify variables in each region of interest (ROI Analyzer; https://github.com/tjrantal/RoiAnalyzer; https://sites.google.com/site/daniellbelavy/home/roianalyser).

#### Intervertebral disc T2, disc height and volume

Per prior work^27^, tracing of each intervertebral disc from T11/T12 to L5/S1 was completed using the sagittal spin-echo sequences (Supplementary Fig. 2). The custom ImageJ plugin rotated the region-of-interest to the horizontal to measure height, width, area, and signal intensity for the whole intervertebral disc and five sub-regions from anterior to posterior. The anatomical slice where the spinous process was most prominent for each subject was noted. To calculate intervertebral disc height (mm), the average height of the three slices of and next to the spinous process were used in our primary analyses and measured in mm. For intervertebral disc volume (reported in cm^3^), we scaled the area of each slice with available data by 4.5 mm to account for the slice thickness and gap between slices. For each disc, T2 (in ms) was calculated by a linear fit of the natural logarithm of the image intensity across the eight echo times and each of the three anatomical slices centred at the spinous process.

#### Vertebral body fat fraction

True-axial Dixon images were used to trace the vertebral bodies and intervertebral discs across the entire lumbar spine (Supplementary Fig. 3)^47^. For each anatomical slice, the signal intensity for both water and fat images was recorded. The fat fraction for each anatomical slice was calculated as:

$$100\% * signal \; intensity \;fat/(signal \;intensity\; fat + signal\; intensity\; water)$$

Following this, at each vertebral level, the average fat fraction was calculated from the three contiguous slices of highest fat fraction. The average fat fraction of all lumbar vertebrae was also calculated.

#### Paraspinal muscle volume, size, and fat fraction

Paraspinal muscles of the multifidus, erector spinae, psoas major and quadratus lumborum were also traced on true-axial Dixon images (Supplementary Fig. 4)^26^. From these, (1) the volume of the left and right paraspinal muscles (from the fifth through to first lumbar level) were calculated by multiplying the area of each slice by 3.5 mm to account for the slice thickness and gap between slices. This value was then transformed into cm^3^ for analyses: (2) For the area of the paraspinal muscles we used the average area of the middle three slices at each level (encompassing the vertebrae and below intervertebral disc to capture the muscle at the lower endplate) and reported in mm^2^: and (3) the fat fraction of the paraspinal muscles across the middle three slices at each level was also calculated using the same equation as for vertebral body fat fraction. The average fat fraction of paraspinal muscles across all lumbar levels was also calculated.

#### Radiographic grading

Radiographic grading of the spinal tissues was completed by a radiographer who was blinded to case–control status (A.T). The intervertebral disc degeneration^48^, facet joint degeneration^49^, pars interarticularis defects^50^, endplate changes (yes/no), interverbal disc osteophyte presence^51^ and herniations^52^ were graded using established approaches. Further details on the radiographic grading criteria are available in Supplementary Table 1.

#### Trunk strength and endurance

Trunk flexion and extension endurance were collected using an established protocol^26,53^. Extension endurance was measured with the participant prone and umbilicus lined up to the edge of the foam mat. Participants lifted their chest, legs and arms off the plinth to a position of neutral spine. Trunk flexion endurance was measured in supine with the hips and knees in a flexed 90/90 position. Participants crossed their arms over their chest and lifted their head and chest off the plinth until the scapula inferior border was no longer in contact. Both positions were held until maximal voluntary fatigue and reported in seconds.

Trunk extension strength was collected using a manual muscle tester (01165-Manual Muscle Tester, Lafayette Instrument Company, Lafayette, United States of America) per an established protocol^54^. Facing forward, participants had their hips strapped to a solid wooden door and with feet placed shoulder width apart. The manual muscle tester was placed between the scapula and the participant was instructed to push as hard as possible against the door behind them. This was conducted three times and maximal extension strength in kg was used.

### Psychosocial questionnaires

The Patient-Reported Outcomes Measurement Information System (PROMIS) framework is a bank of standardised, reliable and validated questionnaires to evaluate physical, mental, and social health in the general population and those with chronic conditions, including pain^15–17^. We used the short-form PROMIS questionnaires for anxiety, depression, cognitive function, general self-efficacy, satisfaction in social roles and activities, social isolation, and social and instrumental support to capture characteristics the psychosocial health domain^15,17^. In addition to the psychosocial questionaries, we examined the presence of other musculoskeletal condition as another co-morbidity driver using the Nordic Musculoskeletal Questionnaire^18^.

### Additional variables used to characterise the collective (not used in data-analytics)

#### Additional questionnaires used to explain sub-groups

We collected self-reported disability using the Oswestry Disability Index^55^ and pain intensity (current, average over prior week and worst over prior week) using the 0–100 mm visual analogue scale^56,57^.

#### Additional demographic data

For additional standard variables, we collected self-report age, ethnicity, education, occupation, employment status, pain duration, handedness, smoking history and use of medications. Quantitative body mass (standard scales, A&D Company Ltd, Tokyo, Japan), body height (standard stadiometer) was collected at the first testing session and body mass index was calculated.

### Statistical and data-analytic methods

**Figure 4:**
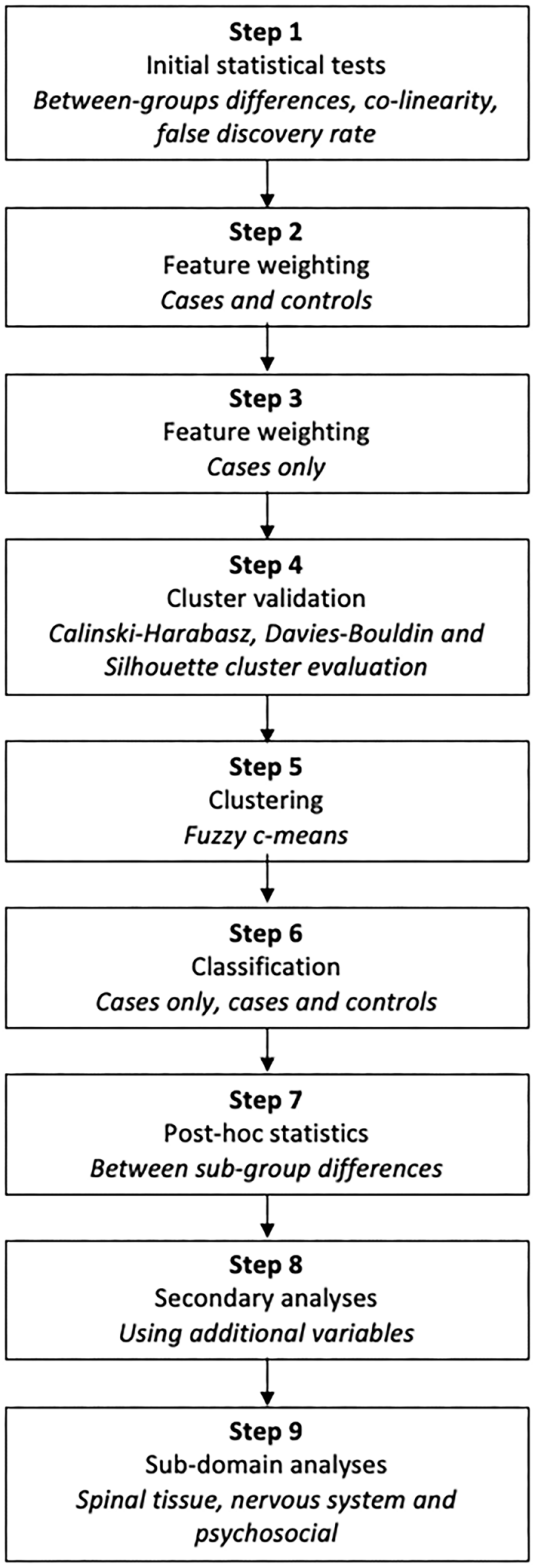
Flow diagram of the analytical pipeline.

All statistical analyses were conducted in R version 4.1.2 (http://www.r-project.org), while MATLAB version R2020a (MathWorks, Massachusetts, USA) was used for data-analytic methods. We followed a standard analytic pipeline reported in our previous publication (Fig. 4), where further details and equations are reported^13^.

*Step 1—Initial statistical tests* Independent t-tests were used to determine between-group differences and explore potentially important variables for data analytic steps. We set an alpha level of 0.05. We used the Benjamini–Hochberg false discovery rate (FDR) method^58^ to adjust p-values to explore which variables may be false positives. However, given the pilot nature of the study, retained significant variables prior to FDR adjustment. Multicollinearity between variables which were significant in t-tests was explored through a correlation matrix of Pearson’s correlation coefficients. We used a threshold of r > 0.8 for determination of collinearity for subsequent steps^59^. Variables passing both of these steps were entered in to data-analytic steps.

*Step 2—Feature weighting between cases and controls* In addition to the independent t-tests, feature weighting was explored to further determine the best variables which separate CLBP and pain-free participants. For this step, data was normalised to a 0–1 scale to allow comparability between variables. We used a random forest predictor to further explore features which helped to differentiate CLBP participants from pain-free controls. Pain-free controls were removed from the data following this step.

*Step 3—Feature ranking in cases only* For variables which made it in to the CLBP only space, Laplacian scores^60^ were used to explore the variables with the most variance and rank them in the order of importance. Laplacian scores are derived from centred (demeaned) pairwise distance metrics within the data space^60^.

*Step 4—Cluster validity* Calinski-Harabasz, Davies-Bouldin and Silhouette cluster evaluation methods with k-means linkage^61^ were used to identify the most appropriate number of clusters which best separated participants with CLBP. To determine the appropriate number of variables to be used in clustering, we repeated the cluster evaluation methods by adding variables into this step in the order of importance determined by the Laplacian scores, until the point where clustering evaluation performance decreased.

*Step 5—Clustering* Fuzzy c-means clustering was then used to derive and label sub-groups of participants with CLBP. We then evaluated the within- and between-cluster distances, as well as the Silhouette index (overall tightness and separation of the data points), to determine how well separated the sub-groups were. We also calculated the discrimination values to determine the density of clusters^62^.

*Step 6—Classification* One-to-one Support Vector Machine (SVM), Naïve Bayes, k-Nearest Neighbour (kNN) and Random Forest (RF) multi-class classifiers were used to determine how accurately the CLBP sub-groups could be classified. Given a tenfold cross validation leads to a biased estimates in small sample sizes, 30 runs of 80/20 train/test holdout split were used, as this has been reported to have unbiased classification accuracy with small samples^63^. Pain-free controls were added back to the main data and classification methods were re-analysed to determine if CLBP sub-groups could be still be accurately classified.

*Step 7—Post-hoc statistical tests* To explore differences between pain-free controls and derived CLBP sub-groups, across both primary and sub-domain analyses, we used analyses of variance (ANOVA). Post-hoc tests with Tukey HSD method for multiple comparisons used to adjust the p-values between groups. We also explored the relationship between variables which dictated clustering and pain intensity and disability within participants with CLBP by calculating the Pearson’s correlation coefficient and 95% confidence intervals.

*Step 8—Secondary analyses* We completed further secondary analyses using the steps above and the additional variables reported in Table 3.

*Step 9—Sub-domain analyses* Given the ability of variables across different domains, and those of a subjective and objective natures, to affect the variance, we explored clustering and classification in each sub-domain of nervous system, spinal tissue and psychosocial separately as a sensitivity analysis using the above methods^4^. The overall classification label was considered across each sub-domain was determined for participants with CLBP.

### Supplementary Information

Supplementary Information.

## Data Availability

The anonymised tabulated data underlying the analyses presented in this study is available via the Open Science Framework (https://osf.io/b4edg/).
