# Supplementary material for: Towards data-driven biopsychosocial classification of non-specific chronic low back pain: a pilot study
Source: Sci Rep. 2023 Aug 12;13:13112. doi: 10.1038/s41598-023-40245-y (PMC10423241; doi:10.1038/s41598-023-40245-y)
Supplement: Supplementary file 1 — Supplementary Information. [file 41598_2023_40245_MOESM1_ESM.docx]

**SUPPLEMENTARY TABLES**

| **Supplementary Table 1.** Radiographic grading criteria | |
| --- | --- |
| *Pfirrmann grading of disc degeneration*^1^ | |
| 1 | Homogenous and bright white structure; clear distinction of the nucleus and anulus; hyperintense, isointense to cerebrospinal fluid; height is normal |
| 2 | Inhomogeneous with or without horizontal bands structure; clear distinction of the nucleus and anulus; hyperintense, isointense to cerebrospinal fluid; height is normal |
| 3 | Inhomogeneous grey structure; unclear distinction between the nucleus and anulus; intermediate signal intensity; normal to slightly decreased height |
| 4 | Inhomogeneous grey to black structure; lost distinction between the nucleus and anulus; intermediate to hypointense signal intensity; normal to moderately decreased height |
| 5 | Inhomogeneous black structure; lost distinction between the nucleus and anulus; hypointense signal intensity; collapsed disc space height |
| *Facet grading criteria*^2^ | |
| 0 | Normal facet joint space (2-4mm width) |
| 1 | Narrowing of the facet joint space (<2mm) and/or small osteophytes and/or mild hypertrophy of the articular process |
| 2 | Narrowing of the facet joint space and/or moderate osteophytes and/or moderate hypertrophy of the articular process and/or subarticular bone erosions |
| 3 | Narrowing of the facet joint space and/or large osteophytes and/or sever hypertrophy of the articular process and/or sever subarticular bone erosions and/or subchondral cysts |
| *Pars defect grading*^3^ | |
| 0: normal | Normal marrow signal; intact cortical margins |
| 1: stress reaction | Marrow oedema; intact cortical margins |
| 2: incomplete fracture | Marrow oedema; cortical fracture incompletely extending through the pars |
| 3: complete active fracture | Marrow oedema; fracture completely extends through the pars |
| 4: fracture non-union | No marrow oedema; fracture complete extends through the pars |
| *Disc bulge grading*^4^ | |
| 0: normal | No disc extension beyond the interspace |
| 1: bulge | Circumferential symmetric extension of the disk beyond the interspace (around the end plates); |
| 2: protrusion | Focal or asymmetric extension of the disk beyond the interspace, with the base against the disk of origin broader than any other dimension of the protrusion |
| 3: extrusion | More extreme extension of the disk beyond the interspace, with the base against the disk of origin narrower than the diameter of the extruding material itself or with no connection between the material and the disk of origin. |
| *Osteophyte grading*^5^ | |
| 0 | None of equivocal |
| 1 | Mild (approx. 1.5 ± 1 mm) |
| 2 | Moderate (approx. 3.5 ± 1 mm) |
| 3 | Severe (approx. ≥ 4.5) |
| *Endplate changes* | |
| Yes/No | Only entered as yes/no on T2 sagittal images due to the lack of T1 images to further classify Modic changes. |

| **Supplementary Table 2.** Chronic low back pain and control participant matching criteria demographics. | | | | | | | | | | | | |
| --- | --- | --- | --- | --- | --- | --- | --- | --- | --- | --- | --- | --- |
| **Participant** | **Sex** | **Age (y)** | **Height (cm)*** | **Body mass index (kg/m^2^)** | **Control** | **Sex** | **Age (y)** | **Height (cm)** | **Body mass index (kg/m^2^)** | **Matched on all criteria?** | **Match on** | **Criteria Difference** |
| CLBP 1 | Male | 50 | 183.2 | 22.5 | Control 1 | Male | 53 | 175.5 | 25.1 | N | Age, sex, BMI | Height: 7.7 |
| CLBP 2 | Male | 37 | 191.5 | 29.6 | Control 2 | Male | 44 | 188.7 | 26.6 | Y | Age, sex, height, BMI | - |
| CLBP 3 | Female | 46 | 169.0 | 29.4 | Control 3 | Female | 50 | 168.9 | 24.2 | Y | Age, sex, height, BMI | - |
| CLBP 4 | Male | 41 | 171.9 | 34.0 | Control 4 | Male | 36 | 174.8 | 25.0 | N | Age, sex height | BMI: 9.0 |
| CLBP 5 | Female | 41 | 170.1 | 30.1 | Control 5 | Female | 38 | 172.4 | 24.4 | N | Age, sex height | BMI: 6.5 |
| CLBP 6 | Male | 31 | 191.5 | 29.6 | Control 6 | Male | 31 | 188.8 | 25.7 | Y | Age, sex, height, BMI | - |
| CLBP 7 | Female | 50 | 160.0 | 40.8 | Control 7 | Female | 50 | 167.7 | 45.5 | N | Age, sex, BMI | Height: 7.7 |
| CLBP 8 | Female | 50 | 166.8 | 23.6 | Control 8 | Female | 46 | 164.3 | 19.0 | Y | Age, sex, height, BMI | - |
| CLBP 9 | Male | 29 | 192.9 | 30.0 | Control 9 | Male | 28 | 192.6 | 23.6 | N | Age, sex height | BMI: 6.4 |
| CLBP 10 | Female | 26 | 158.2 | 24.7 | Control 10 | Female | 25 | 161.8 | 24.3 | Y | Age, sex, height, BMI | - |
| CLBP 11 | Male | 29 | 168.5 | 23.2 | Control 11 | Male | 29 | 171.8 | 27.4 | Y | Age, sex, height, BMI | - |
| CLBP 12 | Female | 50 | 164.7 | 22.5 | Control 12 | Female | 47 | 159.8 | 24.9 | Y | Age, sex, height, BMI | - |
| CLBP 13 | Male | 28 | 187.1 | 20.3 | Control 13 | Male | 31 | 183.8 | 27.4 | N | Age, sex, height | BMI: 7.1 |
| CLBP 14 | Female | 22 | 179.3 | 24.3 | Control 14 | Female | 25 | 172.7 | 25.0 | N | Age, sex, BMI | Height: 7.1 |
| CLBP 15 | Female | 24 | 170.0 | 24.9 | Control 15 | Female | 21 | 166.7 | 22.1 | Y | Age, sex, height, BMI | - |
| CLBP 16 | Female | 20 | 169.9 | 21.4 | Control 16 | Female | 22 | 167.7 | 21.5 | Y | Age, sex, height, BMI | - |
| CLBP 17 | Male | 51 | 173.0 | 33.1 | Control 17 | Male | 51 | 173.0 | 20.1 | N | Age, sex, height | BMI: 13.0 |
| CLBP 18 | Male | 37 | 181.0 | 24.4 | Control 18 | Male | 40 | 179.9 | 23.4 | Y | Age, sex, height, BMI | - |
| CLBP 19 | Male | 21 | 170.8 | 23.5 | Control 19 | Male | 25 | 168.5 | 19.4 | Y | Age, sex, height, BMI | - |
| CLBP 20 | Female | 37 | 165.8 | 29.6 | Control 20 | Female | 37 | 166.7 | 37.0 | N | Age, sex, height | BMI: 7.4 |
| CLBP 21 | Female | 23 | 168.6 | 29.7 | Control 21 | Female | 22 | 165.0 | 19.2 | N | Age, sex, height | BMI: 10.5 |
| CLBP = chronic low back pain, BMI = body mass index  *Height and weight measured collected during telephone screening were used to match participants. Here we report the values measured during testing. | | | | | | | | | | | | |

| **Supplementary Table 3.** T-test results of variables included in the primary analyses | | | | | | | | |
| --- | --- | --- | --- | --- | --- | --- | --- | --- |
| **Variable** | **Pain-free mean** | **Pain-free SD** | **CLBP Mean** | **CLBP SD** | **Mean difference** | **Standardised mean difference (Hedges’ g)** | **p-value** | **Benjamini-Hochberg adjusted p-value** |
| **Number pain sites 12 months** | **1.38** | **1.43** | **5.52** | **2.40** | **-4.1** | **2.07** | **<0.001** | **<0.001** |
| **Number pain sites 7 days** | **0.33** | **0.66** | **2.81** | **1.86** | **-2.5** | **1.88** | **<0.001** | **<0.001** |
| **Central sensitisation inventory** | **17.10** | **10.27** | **36.81** | **14.05** | **-19.7** | **1.55** | **<0.001** | **<0.001** |
| **Social Satisfaction** | **38.05** | **3.83** | **32.38** | **8.59** | **5.7** | **-0.87** | **0.009** | **0.117** |
| **Depressive Symptoms** | **8.76** | **1.26** | **12.10** | **5.98** | **-3.3** | **0.88** | **0.017** | **0.180** |
| **Max extension strength** | **68.71** | **16.22** | **55.36** | **20.80** | **13.4** | **-0.69** | **0.026** | **0.212** |
| **Average lumbar pressure-pain threshold** | **9.05** | **2.17** | **7.39** | **2.62** | **1.7** | **-0.66** | **0.031** | **0.212** |
| **General self-efficacy** | **44.10** | **5.36** | **39.33** | **8.31** | **4.8** | **-0.67** | **0.033** | **0.212** |
| **Anxiety symptoms** | **11.52** | **4.42** | **15.52** | **7.16** | **-4.0** | **0.66** | **0.035** | **0.212** |
| **Cognitive function** | **36.19** | **4.37** | **32.00** | **7.90** | **4.2** | **-0.65** | **0.040** | **0.214** |
| **Lumbar IVD T2-time** | **103.43** | **13.47** | **95.55** | **11.10** | **7.9** | **-0.61** | **0.045** | **0.221** |
| Flexion Endurance | 86.85 | 63.44 | 55.48 | 40.89 | 31.4 | -0.58 | 0.064 | 0.265 |
| Average leg pressure-pain threshold | 7.74 | 2.52 | 6.29 | 2.43 | 1.5 | -0.56 | 0.064 | 0.265 |
| Lumbar QL fat | 15.74 | 3.70 | 17.81 | 3.49 | -2.1 | 0.55 | 0.070 | 0.265 |
| Average Pfirrmann grade | 2.28 | 0.71 | 2.65 | 0.59 | -0.4 | 0.55 | 0.073 | 0.265 |
| Lumbar Psoas fat | 14.57 | 3.59 | 16.28 | 2.81 | -1.7 | 0.51 | 0.093 | 0.315 |
| Average disc bulge | 0.29 | 0.36 | 0.47 | 0.35 | -0.2 | 0.49 | 0.106 | 0.337 |
| Emotional support | 37.14 | 3.80 | 34.52 | 6.58 | 2.6 | -0.48 | 0.122 | 0.356 |
| Social isolation | 12.48 | 4.58 | 15.52 | 7.88 | -3.0 | 0.47 | 0.133 | 0.356 |
| ACC grey matter volume | 11231.49 | 1678.35 | 12166.98 | 2336.99 | -935.5 | 0.45 | 0.144 | 0.356 |
| PCC-AG connectivity | 0.33 | 0.20 | 0.44 | 0.25 | -0.1 | 0.44 | 0.146 | 0.356 |
| Lumbar MF fat | 16.18 | 5.01 | 18.49 | 5.25 | -2.3 | 0.43 | 0.153 | 0.356 |
| Average facet grade | 0.72 | 0.41 | 0.91 | 0.44 | -0.2 | 0.43 | 0.154 | 0.356 |
| Lumbar ES fat | 15.94 | 5.62 | 18.24 | 4.68 | -2.3 | 0.43 | 0.158 | 0.356 |
| Extension endurance | 195.58 | 148.19 | 145.43 | 99.00 | 50.1 | -0.39 | 0.205 | 0.442 |
| Putamen grey matter volume | 5459.06 | 784.49 | 5737.11 | 648.65 | -278.0 | 0.37 | 0.218 | 0.453 |
| Lumbar EIH | 0.86 | 1.30 | 0.41 | 1.13 | 0.5 | -0.35 | 0.238 | 0.477 |
| Lumbar temporal summation | 0.81 | 1.25 | 1.26 | 1.35 | -0.5 | 0.33 | 0.266 | 0.503 |
| Body mass index | 25.28 | 6.05 | 27.20 | 5.03 | -1.9 | 0.33 | 0.270 | 0.503 |
| Hippocampus grey matter volume | 4869.32 | 452.52 | 5014.01 | 468.00 | -144.7 | 0.30 | 0.315 | 0.566 |
| Forearm EIH | 0.60 | 1.26 | 0.25 | 1.24 | 0.4 | -0.27 | 0.370 | 0.644 |
| Leg temporal summation | 1.50 | 1.36 | 1.86 | 1.49 | -0.4 | 0.24 | 0.423 | 0.668 |
| Secondary somatosensory grey matter volume | 4033.66 | 711.21 | 4196.50 | 593.83 | -162.8 | 0.24 | 0.425 | 0.668 |
| Primary motor grey matter volume | 29612.36 | 3742.50 | 30714.85 | 5137.65 | -1102.5 | 0.24 | 0.431 | 0.668 |
| Average pars grade | 0.06 | 0.18 | 0.11 | 0.29 | -0.1 | 0.23 | 0.444 | 0.668 |
| Amygdala grey matter volume | 2592.88 | 341.01 | 2678.04 | 373.87 | -85.2 | 0.23 | 0.445 | 0.668 |
| Primary somatosensory grey matter volume | 23485.90 | 3037.33 | 24252.28 | 3702.93 | -766.4 | 0.22 | 0.468 | 0.683 |
| Medial frontal cortex grey matter volume | 4373.74 | 723.15 | 4233.99 | 740.30 | 139.8 | -0.18 | 0.540 | 0.762 |
| Leg EIH | 0.64 | 1.11 | 0.46 | 0.88 | 0.2 | -0.18 | 0.552 | 0.762 |
| Forearm leg pressure-pain threshold | 5.60 | 1.99 | 5.20 | 2.45 | 0.4 | -0.17 | 0.566 | 0.762 |
| Thalamus grey matter volume | 10801.78 | 1617.01 | 10536.36 | 1452.43 | 265.4 | -0.17 | 0.579 | 0.762 |
| Forearm temporal summation | 1.60 | 1.42 | 1.43 | 1.59 | 0.2 | -0.11 | 0.722 | 0.917 |
| MPFC-NAC connectivity | 0.12 | 0.11 | 0.11 | 0.09 | 0.0 | -0.10 | 0.741 | 0.917 |
| Psoas volume | 149.05 | 51.71 | 153.51 | 44.60 | -4.5 | 0.09 | 0.766 | 0.917 |
| Lumbar vertebrae fat fraction | 53.00 | 10.11 | 53.92 | 10.22 | -0.9 | 0.09 | 0.773 | 0.917 |
| QL volume | 49.90 | 18.30 | 48.54 | 12.74 | 1.4 | -0.08 | 0.781 | 0.917 |
| IVD height | 9.09 | 1.08 | 9.02 | 0.99 | 0.1 | -0.07 | 0.825 | 0.931 |
| Lumbar ES volume | 257.51 | 76.38 | 262.21 | 65.48 | -4.7 | 0.06 | 0.832 | 0.931 |
| Lumbar IVD volume | 11.30 | 3.12 | 11.47 | 2.47 | -0.2 | 0.06 | 0.848 | 0.931 |
| Supplementary motor grey matter volume | 6460.00 | 1269.20 | 6409.79 | 1019.16 | 50.2 | -0.04 | 0.888 | 0.931 |
| Instrumental support | 33.48 | 10.19 | 33.10 | 8.43 | 0.4 | -0.04 | 0.896 | 0.931 |
| Lumbar MF volume | 89.98 | 26.91 | 89.22 | 24.74 | 0.8 | -0.03 | 0.924 | 0.931 |
| Insula grey matter volume | 12731.33 | 1361.38 | 12765.70 | 1192.52 | -34.4 | 0.03 | 0.931 | 0.931 |
| Caudate grey matter volume | 4070.67 | 798.49 | 4051.34 | 637.78 | 19.3 | -0.03 | 0.931 | 0.931 |

| **Supplementary Table 4.** Pearson’s correlation coefficients between significant variables. | | | | | | | | | | | |
| --- | --- | --- | --- | --- | --- | --- | --- | --- | --- | --- | --- |
|  | **(1)** | **(2)** | **(3)** | **(4)** | **(5)** | **(6)** | **(7)** | **(8)** | **(9)** | **(10)** | **(11)** |
| **Number pain sites 12 months (1)** | 1 | 0.76 | 0.77 | 0.41 | -0.43 | 0.45 | -0.39 | -0.47 | -0.23 | -0.39 | -0.21 |
| **Number pain sites 7 days (2)** | 0.76 | 1 | 0.67 | 0.35 | -0.44 | 0.46 | -0.46 | -0.48 | -0.26 | -0.37 | -0.3 |
| **Central sensitisation inventory (3)** | 0.77 | 0.67 | 1 | 0.71 | -0.72 | 0.59 | -0.63 | -0.65 | -0.34 | -0.31 | -0.39 |
| **Anxiety symptoms (4)** | 0.41 | 0.35 | 0.71 | 1 | -0.74 | 0.71 | -0.62 | -0.63 | -0.24 | -0.16 | -0.28 |
| **Cognitive function (5)** | -0.43 | -0.44 | -0.72 | -0.74 | 1 | -0.74 | 0.55 | 0.68 | 0.16 | 0.09 | 0.28 |
| **Depressive symptoms (6)** | 0.45 | 0.46 | 0.59 | 0.71 | -0.74 | 1 | -0.41 | -0.66 | -0.18 | -0.03 | -0.38 |
| **Social satisfaction (7)** | -0.39 | -0.46 | -0.63 | -0.62 | 0.55 | -0.41 | 1 | 0.6 | 0.37 | 0.3 | 0.2 |
| **General self-efficacy (8)** | -0.47 | -0.48 | -0.65 | -0.63 | 0.68 | -0.66 | 0.6 | 1 | 0.27 | 0.16 | 0.32 |
| **Extension strength (9)** | -0.23 | -0.26 | -0.34 | -0.24 | 0.16 | -0.18 | 0.37 | 0.27 | 1 | 0.53 | 0.03 |
| **Lumbar IVD T2-time (10)** | -0.39 | -0.37 | -0.31 | -0.16 | 0.09 | -0.03 | 0.3 | 0.16 | 0.53 | 1 | -0.09 |
| **Lumbar pressure-pain threshold (11)** | -0.21 | -0.3 | -0.39 | -0.28 | 0.28 | -0.38 | 0.2 | 0.32 | 0.03 | -0.09 | 1 |

| **Supplementary Table 5.** Cluster validity methods to determine appropriate cluster number for all variables. | | | |
| --- | --- | --- | --- |
| **Cluster Number** | **Calinski-Harabasz*** | **Davies-Bouldin**** | **Sillhouette*** |
| *2-Variables* | | | |
| K=2 | 54.3 | **0.38** | **0.86** |
| K=3 | 73.1 | 0.55 | 0.78 |
| K=4 | 68.6 | 0.45 | 0.78 |
| K=5 | 64.7 | 0.55 | 0.63 |
| K=6 | **74.5** | 0.48 | 0.62 |
| *3-Variables* | | | |
| K=2 | 40.7 | **0.55** | **0.79** |
| K=3 | **45.5** | 0.62 | 0.72 |
| K=4 | 39.5 | 0.67 | 0.67 |
| K=5 | 39.4 | 0.57 | 0.70 |
| K=6 | 38.5 | 0.71 | 0.52 |
| *4-Variables* | | | |
| K=2 | **41.4** | **0.59** | **0.81** |
| K=3 | 34.4 | 0.86 | 0.62 |
| K=4 | 29.9 | 0.93 | 0.57 |
| K=5 | 28.0 | 0.88 | 0.63 |
| K=6 | 27.4 | 0.91 | 0.51 |
| *5-Variables* | | | |
| K=2 | **26.2** | 0.76 | **0.71** |
| K=3 | 22.6 | 0.95 | 0.66 |
| K=4 | 20.2 | 0.76 | 0.55 |
| K=5 | 18.9 | 0.68 | 0.61 |
| K=6 | 19.9 | **0.63** | 0.62 |
| *6-Variables* | | | |
| K=2 | **24.0** | 0.80 | **0.69** |
| K=3 | 19.4 | 0.89 | 0.49 |
| K=4 | 17.4 | 0.96 | 0.47 |
| K=5 | 14.7 | 0.97 | 0.51 |
| K=6 | 15.4 | **0.68** | 0.49 |
| *7-Variables* | | | |
| K=2 | **18.4** | 0.91 | **0.63** |
| K=3 | 14.3 | 1.26 | 0.46 |
| K=4 | 12.8 | 1.06 | 0.40 |
| K=5 | 11.4 | **0.87** | 0.41 |
| K=6 | 10.3 | 1.01 | 0.44 |
| *8-Variables* | | | |
| K=2 | **14.4** | 1.01 | **0.58** |
| K=3 | 10.7 | 1.29 | 0.41 |
| K=4 | 8.5 | 1.16 | 0.35 |
| K=5 | 8.3 | **0.99** | 0.30 |
| K=6 | 8.1 | 1.07 | 0.38 |
| *9-Variables* | | | |
| K=2 | **12.3** | 1.12 | **0.55** |
| K=3 | 8.3 | 1.60 | 0.42 |
| K=4 | 7.6 | 1.34 | 0.35 |
| K=5 | 6.6 | 1.31 | 0.30 |
| K=6 | 7.1 | **1.07** | 0.34 |
| *10-Variables* | | | |
| K=2 | **11.1** | 1.32 | **0.49** |
| K=3 | 8.4 | 1.42 | 0.35 |
| K=4 | 7.6 | 1.28 | 0.26 |
| K=5 | 6.0 | **1.15** | 0.30 |
| K=6 | 6.5 | 1.16 | 0.35 |
| *Higher values for Calinski-Harabasz and Silhouette values indicate the optimal cluster number. **Lower values for Davies-Bouldin values indicate the optimal class number. | | | |

| **Supplementary Table 6.** Average within- and between-cluster distances on a normalised 0-1 scale for the primary analyses | | |
| --- | --- | --- |
|  | Cluster 1 | Cluster 2 |
| *Within-Cluster Distances* | | |
| Distance | 0.28 | 0.40 |
| *Between-Cluster Distances* | | |
| Cluster 1 | 0 | 1.53 |
| Cluster 2 | 1.53 | 0 |

| **Supplementary Table 7.** Misclassification rates (percentage) on the back pain clusters in the primary analyses. | | | | |
| --- | --- | --- | --- | --- |
| **Run** | **SVM** | **Naïve-Bayes** | **kNN** | **Random Forest** |
| 1 | 0 | 0 | 0 | 25 |
| 2 | 25 | 25 | 0 | 25 |
| 3 | 25 | 25 | 25 | 0 |
| 4 | 0 | 0 | 0 | 0 |
| 5 | 25 | 0 | 25 | 0 |
| 6 | 25 | 0 | 25 | 0 |
| 7 | 0 | 25 | 25 | 0 |
| 8 | 0 | 0 | 0 | 25 |
| 9 | 50 | 0 | 25 | 0 |
| 10 | 0 | 25 | 0 | 0 |
| 11 | 25 | 0 | 25 | 0 |
| 12 | 25 | 0 | 0 | 0 |
| 13 | 25 | 0 | 25 | 0 |
| 14 | 0 | 0 | 0 | 0 |
| 15 | 0 | 0 | 25 | 0 |
| 16 | 25 | 0 | 0 | 0 |
| 17 | 0 | 0 | 25 | 0 |
| 18 | 0 | 0 | 25 | 0 |
| 19 | 0 | 0 | 0 | 0 |
| 20 | 0 | 0 | 0 | 0 |
| 21 | 25 | 0 | 0 | 0 |
| 22 | 25 | 0 | 0 | 0 |
| 23 | 25 | 0 | 25 | 25 |
| 24 | 25 | 0 | 25 | 0 |
| 25 | 0 | 0 | 0 | 0 |
| 26 | 25 | 0 | 0 | 25 |
| 27 | 50 | 0 | 25 | 0 |
| 28 | 0 | 25 | 50 | 0 |
| 29 | 0 | 0 | 0 | 25 |
| 30 | 0 | 0 | 0 | 25 |
| **Average (95%CI)** | **14.2 (8.6, 19.8)** | **4.2 (0.8, 7.6)** | **12.5 (7.4, 17.6)** | **5.8 (2.0, 9.7)** |

| **Supplementary Table 8.** Misclassification rates (percentage) on the back pain and pain-free clusters in the primary analyses. | | | | |
| --- | --- | --- | --- | --- |
| **Run** | **SVM** | **Naïve-Bayes** | **kNN** | **Random Forest** |
| 1 | 25 | 12.5 | 50 | 25 |
| 2 | 25 | 37.5 | 12.5 | 50 |
| 3 | 37.5 | 50 | 25 | 12.5 |
| 4 | 25 | 37.5 | 12.5 | 37.5 |
| 5 | 50 | 25 | 37.5 | 12.5 |
| 6 | 25 | 37.5 | 25 | 0 |
| 7 | 37.5 | 25 | 25 | 12.5 |
| 8 | 37.5 | 25 | 37.5 | 25 |
| 9 | 25 | 12.5 | 25 | 37.5 |
| 10 | 37.5 | 12.5 | 37.5 | 25 |
| 11 | 12.5 | 37.5 | 37.5 | 50 |
| 12 | 37.5 | 37.5 | 37.5 | 25 |
| 13 | 12.5 | 12.5 | 25 | 37.5 |
| 14 | 37.5 | 12.5 | 50 | 25 |
| 15 | 12.5 | 25 | 50 | 37.5 |
| 16 | 0 | 25 | 50 | 25 |
| 17 | 25 | 12.5 | 50 | 0 |
| 18 | 0 | 12.5 | 37.5 | 50 |
| 19 | 25 | 12.5 | 37.5 | 62.5 |
| 20 | 25 | 25 | 25 | 12.5 |
| 21 | 12.5 | 12.5 | 37.5 | 50 |
| 22 | 25 | 12.5 | 75 | 25 |
| 23 | 37.5 | 37.5 | 50 | 0 |
| 24 | 12.5 | 12.5 | 37.5 | 0 |
| 25 | 37.5 | 12.5 | 37.5 | 37.5 |
| 26 | 37.5 | 37.5 | 37.5 | 50 |
| 27 | 37.5 | 25 | 25 | 37.5 |
| 28 | 37.5 | 25 | 50 | 37.5 |
| 29 | 37.5 | 37.5 | 25 | 0 |
| 30 | 12.5 | 25 | 62.5 | 0 |
| **Average (95%CI)** | **26.7 (22.2, 31.2)** | **24.2 (20.1, 28.2)** | **37.5 (32.5, 42.5)** | **26.7 (20.0, 33.3)** |

| **Supplementary Table 9.** T-test results of variables included in the secondary analyses | | | | | | | | |
| --- | --- | --- | --- | --- | --- | --- | --- | --- |
| **Variable** | **Pain-free mean** | **Pain-free SD** | **CLBP Mean** | **CLBP SD** | **Mean difference** | **Standardised mean difference (Hedges’ g)** | **p-value** | **Benjamini-Hochberg adjusted p-value** |
| **Pain sites 12 months** | **1.38** | **1.43** | **5.52** | **2.40** | **-4.14** | **2.07** | **<0.001** | **<0.001** |
| **Pain sites 7 days** | **0.33** | **0.66** | **2.81** | **1.86** | **-2.48** | **1.88** | **<0.001** | **<0.001** |
| **Central sensitisation Inventory** | **17.10** | **10.27** | **36.81** | **14.05** | **-19.71** | **1.55** | **<0.001** | **<0.001** |
| **L5S1 T2-time mid 3 slices** | **99.82** | **13.46** | **85.53** | **14.54** | **14.30** | **-0.98** | **0.002** | **0.066** |
| **L5S1 T2-time nucleus only** | **103.29** | **17.02** | **86.63** | **17.70** | **16.67** | **-0.92** | **0.003** | **0.090** |
| **L4L5 T2-time whole disc** | **90.41** | **11.28** | **80.16** | **11.88** | **10.25** | **-0.85** | **0.007** | **0.143** |
| **Social Satisfaction** | **38.05** | **3.83** | **32.38** | **8.59** | **5.67** | **-0.87** | **0.009** | **0.147** |
| **L4L5 T2-time mid 3 slices** | **103.88** | **17.26** | **87.85** | **20.41** | **16.03** | **-0.81** | **0.009** | **0.147** |
| **L5S1 T2-time whole disc** | **91.11** | **9.72** | **82.21** | **11.73** | **8.90** | **-0.79** | **0.011** | **0.156** |
| **Max Facet Joint Grade** | **1.24** | **0.70** | **1.81** | **0.75** | **-0.57** | **0.75** | **0.015** | **0.191** |
| **Depressive Symptoms** | **8.76** | **1.26** | **12.10** | **5.98** | **-3.33** | **0.88** | **0.017** | **0.199** |
| **L4L5 T2-time nucleus only** | **110.46** | **22.41** | **91.28** | **28.41** | **19.18** | **-0.72** | **0.020** | **0.215** |
| **Max Pfirrmann Grade** | **3.05** | **0.86** | **3.62** | **0.67** | **-0.57** | **0.71** | **0.021** | **0.215** |
| **Max Extension Strength** | **68.71** | **16.22** | **55.36** | **20.80** | **13.35** | **-0.69** | **0.026** | **0.239** |
| **L2 QL fat %** | **14.83** | **4.41** | **17.49** | **3.01** | **-2.66** | **0.69** | **0.028** | **0.241** |
| **Left lumbar pressure-pain threshold** | **9.09** | **2.21** | **7.35** | **2.82** | **1.74** | **-0.66** | **0.032** | **0.244** |
| **General Self-Efficacy** | **44.10** | **5.36** | **39.33** | **8.31** | **4.76** | **-0.67** | **0.033** | **0.244** |
| **Left leg pressure-pain threshold** | **7.80** | **2.50** | **6.12** | **2.50** | **1.68** | **-0.64** | **0.035** | **0.244** |
| **Anxiety** | **11.52** | **4.42** | **15.52** | **7.16** | **-4.00** | **0.66** | **0.035** | **0.244** |
| **Cognitive Function** | **36.19** | **4.37** | **32.00** | **7.90** | **4.19** | **-0.65** | **0.040** | **0.250** |
| **Right lumbar pressure-pain threshold** | **9.01** | **2.24** | **7.42** | **2.59** | **1.59** | **-0.63** | **0.040** | **0.250** |
| L5 ES fat %* | 23.22 | - | 26.31 | - | -3.09 | - | 0.044 | 0.262 |
| L1 QL fat %* | 17.58 | - | 20.71 | - | -3.13 | - | 0.049 | 0.281 |
| L2 PS fat % | 15.19 | 3.99 | 17.31 | 3.16 | -2.12 | 0.57 | 0.063 | 0.336 |
| Trunk flexion endurance | 86.85 | 63.44 | 55.48 | 40.89 | 31.37 | -0.58 | 0.064 | 0.336 |
| L2 MF fat % | 16.22 | 4.71 | 19.15 | 5.64 | -2.92 | 0.54 | 0.076 | 0.381 |
| L4 PS fat % | 13.88 | 3.28 | 15.48 | 2.60 | -1.60 | 0.52 | 0.087 | 0.421 |
| L3 QL fat % | 14.65 | 3.52 | 16.52 | 3.51 | -1.87 | 0.51 | 0.093 | 0.436 |
| Right ACC grey matter volume | 6015.59 | 1051.54 | 6650.82 | 1348.66 | -635.23 | 0.51 | 0.096 | 0.436 |
| Right lumbar exercise induced hypoalgesia | 0.95 | 1.32 | 0.27 | 1.27 | 0.67 | -0.50 | 0.100 | 0.436 |
| L3 PS fat % | 15.23 | 4.00 | 17.03 | 2.95 | -1.79 | 0.49 | 0.106 | 0.448 |
| LF MF fat % | 17.47 | 6.09 | 20.56 | 6.20 | -3.09 | 0.48 | 0.111 | 0.455 |
| Emotional support | 37.14 | 3.80 | 34.52 | 6.58 | 2.62 | -0.48 | 0.122 | 0.472 |
| Right leg pressure-pain threshold | 7.69 | 2.62 | 6.46 | 2.44 | 1.22 | -0.46 | 0.125 | 0.472 |
| L1 ES fat % | 13.04 | 4.81 | 15.11 | 3.70 | -2.07 | 0.46 | 0.127 | 0.472 |
| L1 PS fat % | 18.19 | - | 20.77 | - | -2.58 | - | 0.131 | 0.472 |
| Social Isolation | 12.48 | 4.58 | 15.52 | 7.88 | -3.05 | 0.47 | 0.133 | 0.472 |
| PCC-AG connectivity | 0.33 | 0.20 | 0.44 | 0.25 | -0.10 | 0.44 | 0.146 | 0.499 |
| L3 MF Fat % | 16.61 | 4.73 | 18.99 | 5.76 | -2.38 | 0.43 | 0.151 | 0.499 |
| Right Putamen volume | 2551.84 | 379.12 | 2717.09 | 354.36 | -165.25 | 0.43 | 0.152 | 0.499 |
| L3 ES Fat % | 15.01 | 5.88 | 17.31 | 4.92 | -2.30 | 0.41 | 0.177 | 0.566 |
| Max disc bulge grade | 1.00 | 1.00 | 1.38 | 0.80 | -0.38 | 0.40 | 0.181 | 0.566 |
| Left forearm EIH | 0.58 | 1.41 | -0.07 | 1.76 | 0.64 | -0.39 | 0.198 | 0.592 |
| L2 ES fat % | 13.49 | 5.64 | 15.52 | 4.34 | -2.03 | 0.39 | 0.199 | 0.592 |
| Extension Endurance | 195.58 | 148.19 | 145.43 | 99.00 | 50.14 | -0.39 | 0.205 | 0.596 |
| L5 PS fat % | 13.18 | 3.38 | 14.42 | 3.10 | -1.25 | 0.37 | 0.220 | 0.627 |
| L1L2 T2-time whole disc | 96.50 | 12.62 | 92.20 | 10.69 | 4.31 | -0.35 | 0.240 | 0.669 |
| Left primary motor cortex volume | 14558.92 | 1743.63 | 15399.79 | 2824.16 | -840.87 | 0.35 | 0.253 | 0.680 |
| L4 ES fat % | 16.08 | 6.43 | 18.28 | 5.90 | -2.20 | 0.34 | 0.254 | 0.680 |
| L4 MF fat % | 17.20 | 4.91 | 18.88 | 4.64 | -1.68 | 0.34 | 0.261 | 0.684 |
| Body mass index | 25.28 | 6.05 | 27.20 | 5.03 | -1.92 | 0.33 | 0.270 | 0.694 |
| Left lumbar temporal summation | 0.86 | 1.20 | 1.33 | 1.59 | -0.48 | 0.33 | 0.279 | 0.697 |
| L1L2 T2-Time (mid 3 slices) | 111.89 | 19.72 | 105.47 | 18.40 | 6.42 | -0.32 | 0.282 | 0.697 |
| Left ACC grey matter volume | 5215.90 | 728.49 | 5516.16 | 1056.81 | -300.26 | 0.32 | 0.290 | 0.704 |
| Right medial frontal cortex volume | 2288.02 | 375.70 | 2162.39 | 402.46 | 125.63 | -0.31 | 0.302 | 0.719 |
| Right leg EIH | 0.66 | 1.18 | 0.32 | 0.95 | 0.34 | -0.30 | 0.315 | 0.725 |
| Right amygdala volume | 1202.69 | 169.29 | 1261.75 | 212.39 | -59.06 | 0.30 | 0.325 | 0.725 |
| Left forearm temporal summation | 1.81 | 1.50 | 1.29 | 1.90 | 0.52 | -0.29 | 0.328 | 0.725 |
| Left hippocampus grey matter volume | 2572.43 | 231.11 | 2646.69 | 257.62 | -74.26 | 0.29 | 0.331 | 0.725 |
| Left putamen grey matter volume | 2907.23 | 420.42 | 3020.02 | 316.41 | -112.80 | 0.29 | 0.332 | 0.725 |
| Right leg temporal summation | 1.71 | 1.65 | 2.19 | 1.63 | -0.48 | 0.28 | 0.352 | 0.754 |
| Right hippocampus grey matter volume | 2296.89 | 260.06 | 2367.32 | 230.80 | -70.43 | 0.27 | 0.359 | 0.754 |
| Right lumbar temporal summation | 0.76 | 1.58 | 1.19 | 1.44 | -0.43 | 0.27 | 0.363 | 0.754 |
| L1L2 T2-time (mid 3 slices) | 104.55 | 15.10 | 100.70 | 12.81 | 3.85 | -0.26 | 0.378 | 0.774 |
| L5 MF Fat % | 17.13 | 5.62 | 18.49 | 4.68 | -1.36 | 0.25 | 0.399 | 0.803 |
| Left primary somatosensory grey matter volume | 12469.88 | 1621.30 | 12949.33 | 2137.72 | -479.46 | 0.24 | 0.418 | 0.817 |
| LF PS Area | 1252.02 | 347.13 | 1328.65 | 281.12 | -76.63 | 0.23 | 0.436 | 0.817 |
| Left forearm pressure-pain threshold | 5.72 | 2.06 | 5.18 | 2.46 | 0.54 | -0.23 | 0.442 | 0.817 |
| L5 ES area | 514.53 | - | 449.08 | - | 65.45 | - | 0.442 | 0.817 |
| L2L3 IVD Volume | 11.28 | 3.29 | 12.03 | 2.98 | -0.75 | 0.23 | 0.444 | 0.817 |
| Max Pars grade | 0.29 | 0.90 | 0.57 | 1.43 | -0.29 | 0.23 | 0.444 | 0.817 |
| L5S1 IVD Height | 9.34 | 1.46 | 9.00 | 1.42 | 0.34 | -0.23 | 0.449 | 0.817 |
| Left Secondary somatosensory cortex grey matter volume volume | 2072.01 | 406.95 | 2162.69 | 378.55 | -90.68 | 0.22 | 0.459 | 0.824 |
| Left thalamus grey matter volume | 5671.08 | 822.05 | 5515.41 | 712.43 | 155.67 | -0.19 | 0.516 | 0.912 |
| Right Secondary somatosensory cortex grey matter volume volume | 1961.65 | 393.26 | 2033.81 | 343.72 | -72.16 | 0.19 | 0.530 | 0.912 |
| L2L3 IVD height | 8.51 | 0.99 | 8.75 | 1.45 | -0.24 | 0.19 | 0.533 | 0.912 |
| L2 ES area | 1903.82 | 503.65 | 1992.78 | 438.92 | -88.96 | 0.18 | 0.545 | 0.912 |
| L3L4 T2-Time Nucleus only | 113.28 | 21.39 | 109.34 | 21.31 | 3.94 | -0.18 | 0.554 | 0.912 |
| L2L3 T2-Time Nucleus only | 110.93 | 22.32 | 106.98 | 20.76 | 3.94 | -0.18 | 0.557 | 0.912 |
| L2L3 T2-time (whole IVD) | 93.83 | 14.20 | 91.49 | 11.32 | 2.35 | -0.18 | 0.557 | 0.912 |
| Left Lumbar EIH | 0.78 | 1.38 | 0.55 | 1.33 | 0.23 | -0.16 | 0.591 | 0.931 |
| L2L3 T2-Time (mid 3 slices) | 103.78 | 17.21 | 101.11 | 15.48 | 2.67 | -0.16 | 0.599 | 0.931 |
| L3L T2-Time (mid 3 slices) | 105.12 | 16.00 | 102.56 | 15.36 | 2.56 | -0.16 | 0.600 | 0.931 |
| Right primary somatosensory cortex grey matter volume | 11016.03 | 1703.52 | 11302.94 | 1863.29 | -286.92 | 0.15 | 0.605 | 0.931 |
| Left leg temporal summation | 1.29 | 1.45 | 1.52 | 1.57 | -0.24 | 0.15 | 0.613 | 0.931 |
| L1 fat fraction | 49.48 | 9.65 | 50.99 | 9.92 | -1.52 | 0.15 | 0.619 | 0.931 |
| L4 PS area | 1301.21 | 407.07 | 1356.35 | 296.67 | -55.14 | 0.15 | 0.619 | 0.931 |
| L2 fat fraction | 51.25 | 10.32 | 52.78 | 10.03 | -1.53 | 0.14 | 0.629 | 0.931 |
| L1 MF area | 233.27 | 64.05 | 223.70 | 64.63 | 9.57 | -0.14 | 0.632 | 0.931 |
| L4 QL fat % | 17.18 | 6.27 | 17.98 | 4.64 | -0.80 | 0.14 | 0.642 | 0.931 |
| Left amygdala grey matter volume | 1390.19 | 195.06 | 1416.30 | 178.16 | -26.11 | 0.13 | 0.653 | 0.931 |
| Right thalamus grey matter volume | 5130.70 | 806.00 | 5020.95 | 769.13 | 109.75 | -0.13 | 0.654 | 0.931 |
| L3 ES area | 1743.35 | 465.20 | 1801.63 | 410.96 | -58.28 | 0.13 | 0.669 | 0.943 |
| L4L5 IVD height | 10.31 | 1.79 | 10.12 | 1.27 | 0.19 | -0.12 | 0.693 | 0.943 |
| Right forearm temporal summation | 1.38 | 1.53 | 1.57 | 1.63 | -0.19 | 0.12 | 0.698 | 0.943 |
| L3 MF area | 473.72 | 140.52 | 489.05 | 140.07 | -15.32 | 0.10 | 0.725 | 0.943 |
| L3L4 IVD height | 9.81 | 1.31 | 9.68 | 1.18 | 0.13 | -0.10 | 0.730 | 0.943 |
| Right forearm pressure-pain threshold | 5.48 | 2.00 | 5.23 | 2.72 | 0.25 | -0.10 | 0.731 | 0.943 |
| Left supplementary motor cortex grey matter volume | 3179.65 | 654.63 | 3117.71 | 499.68 | 61.93 | -0.10 | 0.732 | 0.943 |
| L3L4 T2-Time (whole IVD) | 92.13 | 11.54 | 91.00 | 10.00 | 1.13 | -0.10 | 0.736 | 0.943 |
| Right primary motor cortex volume | 15053.44 | 2350.24 | 15315.07 | 2655.16 | -261.62 | 0.10 | 0.737 | 0.943 |
| L1L2 IVD volume | 9.48 | 2.72 | 9.73 | 2.04 | -0.25 | 0.10 | 0.740 | 0.943 |
| mPFC-nAC connectivity | 0.12 | 0.11 | 0.11 | 0.09 | 0.01 | -0.10 | 0.741 | 0.943 |
| Right caudate primary motor cortex volume | 1895.52 | 402.96 | 1859.25 | 332.67 | 36.27 | -0.09 | 0.752 | 0.947 |
| L1 ES area | 1817.54 | 481.52 | 1860.47 | 494.11 | -42.94 | 0.08 | 0.777 | 0.957 |
| L4 ES area | 1333.34 | 377.06 | 1362.51 | 285.40 | -29.17 | 0.08 | 0.779 | 0.957 |
| L2 MF area | 321.43 | 78.70 | 313.38 | 106.47 | 8.05 | -0.08 | 0.782 | 0.957 |
| L5 MF area | 854.63 | 227.29 | 871.16 | 187.12 | -16.53 | 0.08 | 0.798 | 0.961 |
| L3 PS area | 971.08 | 329.27 | 995.44 | 300.11 | -24.36 | 0.07 | 0.803 | 0.961 |
| L2 QL area | 282.19 | 139.49 | 290.48 | 94.49 | -8.29 | 0.07 | 0.823 | 0.961 |
| L3 fat fraction | 54.30 | 10.29 | 55.00 | 10.08 | -0.71 | 0.07 | 0.824 | 0.961 |
| L4 fat fraction | 54.97 | 10.80 | 55.70 | 10.74 | -0.73 | 0.06 | 0.827 | 0.961 |
| L2 PS area | 566.97 | 224.42 | 581.39 | 223.11 | -14.42 | 0.06 | 0.836 | 0.961 |
| L1L2 IVD height | 7.46 | 1.07 | 7.53 | 1.04 | -0.07 | 0.06 | 0.838 | 0.961 |
| L4 MF area | 785.72 | 210.72 | 772.04 | 234.71 | 13.68 | -0.06 | 0.844 | 0.961 |
| Right insula grey matter volume | 6371.25 | 734.87 | 6411.41 | 633.98 | -40.16 | 0.06 | 0.851 | 0.961 |
| L1 PS area | 234.94 | - | 241.40 | - | -6.46 | - | 0.869 | 0.967 |
| L1 QL area | 176.91 | - | 173.54 | - | 3.37 | - | 0.880 | 0.967 |
| Right forearm EIH | 0.63 | 1.29 | 0.58 | 1.26 | 0.06 | -0.04 | 0.885 | 0.967 |
| Left caudate grey matter volume | 2175.15 | 425.29 | 2192.08 | 342.17 | -16.93 | 0.04 | 0.888 | 0.967 |
| Instrumental support | 33.48 | 10.19 | 33.10 | 8.43 | 0.38 | -0.04 | 0.896 | 0.967 |
| Left frontomedial cortex grey matter volume | 2085.72 | 363.75 | 2071.60 | 364.87 | 14.12 | -0.04 | 0.901 | 0.967 |
| L5S1 IVD Volume | 9.35 | 2.53 | 9.26 | 2.48 | 0.09 | -0.03 | 0.909 | 0.968 |
| Left leg EIH | 0.63 | 1.37 | 0.59 | 0.98 | 0.04 | -0.03 | 0.923 | 0.975 |
| L4 QL area | 571.49 | 172.01 | 575.38 | 137.34 | -3.88 | 0.02 | 0.936 | 0.980 |
| Right supplementary motor cortex grey matter volume | 3280.36 | 673.68 | 3292.07 | 571.85 | -11.72 | 0.02 | 0.952 | 0.980 |
| L3 QL area | 436.20 | 177.04 | 438.27 | 147.68 | -2.07 | 0.01 | 0.967 | 0.980 |
| L4L5 IVD Volume | 13.25 | 4.20 | 13.21 | 2.95 | 0.04 | -0.01 | 0.974 | 0.980 |
| Left insula grey matter volume | 6360.08 | 643.32 | 6354.29 | 586.22 | 5.79 | -0.01 | 0.976 | 0.980 |
| L3L4 IVD volume | 13.14 | 4.03 | 13.11 | 3.13 | 0.03 | -0.01 | 0.977 | 0.980 |
| L5 fat fraction | 55.02 | 10.13 | 55.10 | 11.18 | -0.08 | 0.01 | 0.980 | 0.980 |
| *unable to be used in data-analytic methods due to missing data across the middle three slices at these levels due to anatomical differences. P-values were calculated on the available data. | | | | | | | | |

| **Supplementary Table 10.** Pearson’s correlation coefficients between significant variables in secondary analyses. | | | | | | | | | | | | | | | | | | | | | |
| --- | --- | --- | --- | --- | --- | --- | --- | --- | --- | --- | --- | --- | --- | --- | --- | --- | --- | --- | --- | --- | --- |
|  | **1** | **2** | **3** | **4** | **5** | **6** | **7** | **8** | **9** | **10** | **11** | **12** | **13** | **14** | **15** | **16** | **17** | **18** | **19** | **20** | **21** |
| 1. **Pain sites 12 months** | 1 | 0.76 | 0.77 | 0.41 | -0.43 | 0.45 | -0.39 | -0.47 | -0.34 | -0.36 | -0.34 | -0.44 | -0.45 | -0.41 | 0.28 | -0.23 | -0.19 | -0.3 | 0.24 | 0.08 | -0.23 |
| 1. **Pain sites 7 days** | 0.76 | 1 | 0.67 | 0.35 | -0.44 | 0.46 | -0.46 | -0.48 | -0.27 | -0.29 | -0.28 | -0.44 | -0.44 | -0.4 | 0.32 | -0.33 | -0.25 | -0.37 | 0.21 | 0.25 | -0.26 |
| 1. **Central sensitisation Inventory** | 0.77 | 0.67 | 1 | 0.71 | -0.72 | 0.59 | -0.63 | -0.65 | -0.32 | -0.36 | -0.37 | -0.26 | -0.26 | -0.23 | 0.37 | -0.39 | -0.37 | -0.38 | 0.15 | 0.19 | -0.34 |
| 1. **Anxiety** | 0.41 | 0.35 | 0.71 | 1 | -0.74 | 0.71 | -0.62 | -0.63 | -0.32 | -0.35 | -0.36 | -0.1 | -0.09 | -0.06 | 0.24 | -0.27 | -0.27 | -0.25 | 0.21 | 0.06 | -0.24 |
| 1. **Cognitive Function** | -0.43 | -0.44 | -0.72 | -0.74 | 1 | -0.74 | 0.55 | 0.68 | 0.22 | 0.25 | 0.27 | 0.01 | 0.03 | 0 | -0.21 | 0.28 | 0.26 | 0.32 | -0.17 | -0.18 | 0.16 |
| 1. **Depressive Symptoms** | 0.45 | 0.46 | 0.59 | 0.71 | -0.74 | 1 | -0.41 | -0.66 | -0.29 | -0.28 | -0.27 | -0.05 | -0.04 | -0.01 | 0.18 | -0.37 | -0.36 | -0.36 | 0.12 | 0.22 | -0.18 |
| 1. **Social Satisfaction** | -0.39 | -0.46 | -0.63 | -0.62 | 0.55 | -0.41 | 1 | 0.6 | 0.13 | 0.23 | 0.23 | 0.21 | 0.28 | 0.27 | -0.33 | 0.22 | 0.18 | 0.22 | -0.16 | -0.1 | 0.37 |
| 1. **General Self-Efficacy** | -0.47 | -0.48 | -0.65 | -0.63 | 0.68 | -0.66 | 0.6 | 1 | 0.17 | 0.22 | 0.22 | 0.03 | 0.09 | 0.08 | -0.2 | 0.33 | 0.29 | 0.39 | -0.16 | -0.05 | 0.27 |
| 1. **L5S1 T2-time whole disc** | -0.34 | -0.27 | -0.32 | -0.32 | 0.22 | -0.29 | 0.13 | 0.17 | 1 | 0.96 | 0.94 | 0.31 | 0.28 | 0.28 | -0.36 | 0.06 | 0.01 | -0.07 | -0.4 | -0.31 | 0.26 |
| 1. **L5S1 T2-time mid 3 slices** | -0.36 | -0.29 | -0.36 | -0.35 | 0.25 | -0.28 | 0.23 | 0.22 | 0.96 | 1 | 0.99 | 0.3 | 0.3 | 0.3 | -0.41 | 0.05 | 0 | -0.09 | -0.52 | -0.33 | 0.32 |
| 1. **L5S1 T2-time nucleus only** | -0.34 | -0.28 | -0.37 | -0.36 | 0.27 | -0.27 | 0.23 | 0.22 | 0.94 | 0.99 | 1 | 0.26 | 0.27 | 0.27 | -0.42 | 0.05 | 0.01 | -0.1 | -0.55 | -0.33 | 0.3 |
| 1. **L4L5 T2-time whole disc** | -0.44 | -0.44 | -0.26 | -0.1 | 0.01 | -0.05 | 0.21 | 0.03 | 0.31 | 0.3 | 0.26 | 1 | 0.95 | 0.94 | -0.45 | 0.06 | -0.01 | 0.05 | -0.24 | -0.37 | 0.3 |
| 1. **L4L5 T2-time mid 3 slices** | -0.45 | -0.44 | -0.26 | -0.09 | 0.03 | -0.04 | 0.28 | 0.09 | 0.28 | 0.3 | 0.27 | 0.95 | 1 | 0.99 | -0.52 | 0.06 | -0.01 | 0.07 | -0.3 | -0.39 | 0.35 |
| 1. **L4L5 T2-time nucleus only** | -0.41 | -0.4 | -0.23 | -0.06 | 0 | -0.01 | 0.27 | 0.08 | 0.28 | 0.3 | 0.27 | 0.94 | 0.99 | 1 | -0.52 | 0.02 | -0.04 | 0.04 | -0.31 | -0.38 | 0.36 |
| 1. **L2 QL fat %** | 0.28 | 0.32 | 0.37 | 0.24 | -0.21 | 0.18 | -0.33 | -0.2 | -0.36 | -0.41 | -0.42 | -0.45 | -0.52 | -0.52 | 1 | -0.12 | -0.18 | -0.17 | 0.25 | 0.54 | -0.5 |
| 1. **Left lumbar pressure-pain threshold** | -0.23 | -0.33 | -0.39 | -0.27 | 0.28 | -0.37 | 0.22 | 0.33 | 0.06 | 0.05 | 0.05 | 0.06 | 0.06 | 0.02 | -0.12 | 1 | 0.9 | 0.81 | 0.08 | -0.13 | 0.02 |
| 1. **Right lumbar pressure-pain threshold** | -0.19 | -0.25 | -0.37 | -0.27 | 0.26 | -0.36 | 0.18 | 0.29 | 0.01 | 0 | 0.01 | -0.01 | -0.01 | -0.04 | -0.18 | 0.9 | 1 | 0.82 | 0.08 | -0.17 | 0.04 |
| 1. **Left leg pressure-pain threshold** | -0.3 | -0.37 | -0.38 | -0.25 | 0.32 | -0.36 | 0.22 | 0.39 | -0.07 | -0.09 | -0.1 | 0.05 | 0.07 | 0.04 | -0.17 | 0.81 | 0.82 | 1 | 0.08 | -0.1 | 0.13 |
| 1. **Max Pfirrmann Grade** | 0.24 | 0.21 | 0.15 | 0.21 | -0.17 | 0.12 | -0.16 | -0.16 | -0.4 | -0.52 | -0.55 | -0.24 | -0.3 | -0.31 | 0.25 | 0.08 | 0.08 | 0.08 | 1 | 0.3 | -0.12 |
| 1. **Max Facet Joint Grade** | 0.08 | 0.25 | 0.19 | 0.06 | -0.18 | 0.22 | -0.1 | -0.05 | -0.31 | -0.33 | -0.33 | -0.37 | -0.39 | -0.38 | 0.54 | -0.13 | -0.17 | -0.1 | 0.3 | 1 | -0.39 |
| 1. **Max Extension Strength** | -0.23 | -0.26 | -0.34 | -0.24 | 0.16 | -0.18 | 0.37 | 0.27 | 0.26 | 0.32 | 0.3 | 0.3 | 0.35 | 0.36 | -0.5 | 0.02 | 0.04 | 0.13 | -0.12 | -0.39 | 1 |

| **Supplementary Table 11.** Cluster validity methods to determine appropriate cluster number for all variables in the secondary analyses. | | | |
| --- | --- | --- | --- |
| **Cluster Number** | **Calinski-Harabasz*** | **Davies-Bouldin**** | **Sillhouette*** |
| *2-Variables* | | | |
| K=2 | 54.3 | **0.38** | **0.86** |
| K=3 | 73.1 | 0.55 | 0.78 |
| K=4 | 68.6 | 0.45 | 0.78 |
| K=5 | 64.7 | 0.55 | 0.63 |
| K=6 | **74.5** | 0.48 | 0.62 |
| *3-Variables* | | | |
| K=2 | **50.9** | **0.54** | **0.84** |
| K=3 | 39.1 | 0.81 | 0.62 |
| K=4 | 35.3 | 0.84 | 0.55 |
| K=5 | 33.2 | 0.73 | 0.52 |
| K=6 | 32.5 | 0.64 | 0.50 |
| *4-Variables* | | | |
| K=2 | 17.8 | 0.80 | **0.67** |
| K=3 | **24.4** | **0.74** | 0.64 |
| K=4 | 23.0 | 0.74 | 0.63 |
| K=5 | 20.5 | 0.80 | 0.57 |
| K=6 | 19.7 | 0.80 | 0.57 |
| *5-Variables* | | | |
| K=2 | 18.7 | **0.81** | **0.67** |
| K=3 | **21.1** | 0.83 | 0.58 |
| K=4 | 19.1 | 0.90 | 0.58 |
| K=5 | 15.4 | 0.97 | 0.55 |
| K=6 | 17.9 | 0.88 | 0.51 |
| *6-Variables* | | | |
| K=2 | **16.0** | 0.96 | **0.62** |
| K=3 | 13.7 | 1.01 | 0.48 |
| K=4 | 12.3 | 10.2 | 0.48 |
| K=5 | 11.4 | 1.10 | 0.54 |
| K=6 | 10.8 | **0.86** | 0.47 |
| *7-Variables* | | | |
| K=2 | **15.8** | **0.98** | **0.61** |
| K=3 | 12.4 | 1.01 | 0.45 |
| K=4 | 10.9 | 1.04 | 0.44 |
| K=5 | 10.1 | 1.14 | 0.49 |
| K=6 | 9.6 | 1.06 | 0.46 |
| *8-Variables* | | | |
| K=2 | **13.7** | 1.04 | **0.57** |
| K=3 | 10.9 | 1.17 | 0.41 |
| K=4 | 10.0 | 1.06 | 0.38 |
| K=5 | 9.5 | **0.97** | 0.48 |
| K=6 | 9.1 | 1.14 | 0.46 |
| *9-Variables* | | | |
| K=2 | **12.3** | 1.12 | **0.55** |
| K=3 | 9.7 | 1.35 | 0.40 |
| K=4 | 9.4 | 1.16 | 0.36 |
| K=5 | 8.3 | 1.09 | 0.38 |
| K=6 | 7.6 | **0.95** | 0.49 |
| *10-Variables* | | | |
| K=2 | **10.8** | 1.19 | **0.51** |
| K=3 | 8.1 | 1.38 | 0.32 |
| K=4 | 8.0 | 1.23 | 0.30 |
| K=5 | 7.2 | 1.27 | 0.38 |
| K=6 | 6.7 | **1.07** | 0.35 |
| *11-Variables* | | | |
| K=2 | **9.5** | 1.26 | **0.48** |
| K=3 | 7.0 | 1.52 | 0.31 |
| K=4 | 5.4 | 1.51 | 0.32 |
| K=5 | 4.7 | 1.15 | 0.28 |
| K=6 | 5.3 | **1.13** | 0.34 |
| *12-Variables* | | | |
| K=2 | **9.5** | 1.26 | **0.48** |
| K=3 | 6.9 | 1.52 | 0.34 |
| K=4 | 5.8 | 1.40 | 0.31 |
| K=5 | 5.9 | **1.15** | 0.33 |
| K=6 | 5.4 | 1.30 | 0.28 |
| *13-Variables* | | | |
| K=2 | **7.9** | 1.39 | **0.43** |
| K=3 | 6.1 | 1.70 | 0.27 |
| K=4 | 5.0 | 1.60 | 0.29 |
| K=5 | 5.0 | **1.29** | 0.19 |
| K=6 | 4.1 | 1.37 | 0.26 |
| *14-Variables* | | | |
| K=2 | **7.6** | 1.43 | **0.42** |
| K=3 | 5.4 | 1.76 | 0.22 |
| K=4 | 4.4 | 1.54 | 0.21 |
| K=5 | 4.0 | 1.47 | 0.21 |
| K=6 | 4.2 | **1.42** | 0.26 |
| *Higher values for Calinski-Harabasz and Silhouette values indicate the optimal cluster number. **Lower values for Davies-Bouldin values indicate the optimal class number. | | | |

| **Supplementary Table 12.** Average within- and between-cluster distances on a normalised 0-1 scale for the secondary analyses | | |
| --- | --- | --- |
|  | Cluster 1 | Cluster 2 |
| *Within-Cluster Distances* | | |
| Distance | 0.22 | 0.38 |
| *Between-Cluster Distances (Discrimination value)* | | |
| Cluster 1 | 0 | 1.44 (-2.3) |
| Cluster 2 | 1.44 (-2.3) | 0 |

| **Supplementary Table 13.** Misclassification rates (percentage) on the back pain clusters in the secondary analyses. | | | | |
| --- | --- | --- | --- | --- |
| **Run** | **SVM** | **Naïve-Bayes** | **kNN** | **Random Forest** |
| 1 | 25 | 25 | 0 | 0 |
| 2 | 0 | 0 | 25 | 0 |
| 3 | 25 | 0 | 25 | 0 |
| 4 | 25 | 0 | 25 | 50 |
| 5 | 0 | 25 | 25 | 0 |
| 6 | 0 | 0 | 0 | 0 |
| 7 | 50 | 25 | 25 | 0 |
| 8 | 25 | 25 | 75 | 0 |
| 9 | 25 | 25 | 0 | 25 |
| 10 | 0 | 25 | 25 | 0 |
| 11 | 25 | 0 | 0 | 0 |
| 12 | 0 | 0 | 25 | 0 |
| 13 | 0 | 25 | 50 | 25 |
| 14 | 0 | 25 | 25 | 0 |
| 15 | 0 | 0 | 0 | 0 |
| 16 | 0 | 0 | 25 | 25 |
| 17 | 25 | 0 | 0 | 0 |
| 18 | 0 | 0 | 25 | 0 |
| 19 | 25 | 0 | 25 | 0 |
| 20 | 0 | 0 | 25 | 25 |
| 21 | 0 | 0 | 25 | 0 |
| 22 | 25 | 0 | 0 | 0 |
| 23 | 0 | 0 | 25 | 25 |
| 24 | 0 | 0 | 0 | 0 |
| 25 | 0 | 0 | 25 | 25 |
| 26 | 25 | 0 | 25 | 0 |
| 27 | 0 | 0 | 25 | 25 |
| 28 | 25 | 0 | 0 | 0 |
| 29 | 25 | 0 | 50 | 25 |
| 30 | 0 | 0 | 25 | 25 |
| **Average (95%CI)** | **11.7 (6.6, 16.8)** | **6.7 (2.6, 10.7)** | **20.8 (14.7, 27.1)** | **9.2 (4.2, 14.1)** |

| **Supplementary Table 14.** Misclassification rates (percentage) on the back pain and pain-free clusters in the secondary analyses. | | | | |
| --- | --- | --- | --- | --- |
| **Run** | **SVM** | **Naïve-Bayes** | **kNN** | **Random Forest** |
| 1 | 25 | 37.5 | 37.5 | 25 |
| 2 | 50 | 12.5 | 37.5 | 37.5 |
| 3 | 25 | 12.5 | 50 | 37.5 |
| 4 | 0 | 37.5 | 37.5 | 12.5 |
| 5 | 25 | 25 | 37.5 | 25 |
| 6 | 37.5 | 12.5 | 25 | 37.5 |
| 7 | 12.5 | 12.5 | 25 | 37.5 |
| 8 | 37.5 | 25 | 25 | 37.5 |
| 9 | 50 | 0 | 50 | 50 |
| 10 | 0 | 12.5 | 50 | 37.5 |
| 11 | 37.5 | 12.5 | 25 | 12.5 |
| 12 | 25 | 25 | 37.5 | 25 |
| 13 | 50 | 25 | 50 | 25 |
| 14 | 12.5 | 50 | 12.5 | 37.5 |
| 15 | 37.5 | 12.5 | 0 | 12.5 |
| 16 | 25 | 62.5 | 37.5 | 12.5 |
| 17 | 12.5 | 25 | 37.5 | 25 |
| 18 | 25 | 37.5 | 12.5 | 25 |
| 19 | 37.5 | 12.5 | 12.5 | 12.5 |
| 20 | 50 | 25 | 50 | 62.5 |
| 21 | 37.5 | 75 | 12.5 | 50 |
| 22 | 37.5 | 37.5 | 50 | 25 |
| 23 | 37.5 | 12.5 | 25 | 37.5 |
| 24 | 25 | 12.5 | 12.5 | 37.5 |
| 25 | 50 | 25 | 37.5 | 25 |
| 26 | 50 | 25 | 25 | 0 |
| 27 | 62.5 | 12.5 | 25 | 12.5 |
| 28 | 12.5 | 12.5 | 50 | 37.5 |
| 29 | 62.5 | 12.5 | 37.5 | 25 |
| 30 | 12.5 | 12.5 | 37.5 | 12.5 |
| **Average (95%CI)** | **32.1 (26.0, 38.2)** | **23.8 (17.8, 29.7)** | **32.1 (27.0, 37.2)** | **28.2 (23.4, 33.3)** |

| **Supplementary Table 15.** Cluster validity methods to determine appropriate cluster number for all variables in the sub-domain analyses on spinal tissues only. | | | |
| --- | --- | --- | --- |
| **Cluster Number** | **Calinski-Harabasz*** | **Davies-Bouldin**** | **Sillhouette*** |
| *2-Variables* | | | |
| K=2 | **30.6** | 0.72 | **0.69** |
| K=3 | 28.5 | 0.74 | 0.60 |
| K=4 | 27.2 | 0.74 | 0.49 |
| K=5 | 28.0 | 0.64 | 0.58 |
| K=6 | 28.4 | **0.53** | 0.63 |
| *Higher values for Calinski-Harabasz and Silhouette values indicate the optimal cluster number. **Lower values for Davies-Bouldin values indicate the optimal class number. | | | |

| **Supplementary Table 16.** Average within- and between-cluster distances on a normalised 0-1 scale for the sub-domain analysis on spinal tissues only. | | |
| --- | --- | --- |
|  | Cluster 1 | Cluster 2 |
| *Within-Cluster Distances* | | |
| Distance | 0.20 | 0.23 |
| *Between-Cluster Distances (Discrimination Value)* | | |
| Cluster 1 | 0 | 1.16 (-1.9) |
| Cluster 2 | 1.16 (-1.9) | 0 |

| **Supplementary Table 17.** Misclassification rates (percentage) on the back pain clusters in the sub‑domain analysis on spinal tissues only. | | | | |
| --- | --- | --- | --- | --- |
| **Run** | **SVM** | **Naïve-Bayes** | **kNN** | **Random Forest** |
| 1 | 0 | 0 | 0 | 50 |
| 2 | 0 | 50 | 0 | 25 |
| 3 | 25 | 25 | 0 | 0 |
| 4 | 0 | 0 | 25 | 0 |
| 5 | 25 | 0 | 0 | 0 |
| 6 | 50 | 0 | 0 | 0 |
| 7 | 0 | 0 | 0 | 0 |
| 8 | 25 | 0 | 25 | 50 |
| 9 | 25 | 25 | 0 | 50 |
| 10 | 25 | 50 | 0 | 0 |
| 11 | 25 | 0 | 50 | 25 |
| 12 | 25 | 0 | 0 | 0 |
| 13 | 0 | 0 | 25 | 50 |
| 14 | 50 | 0 | 0 | 0 |
| 15 | 25 | 25 | 0 | 25 |
| 16 | 0 | 0 | 0 | 25 |
| 17 | 25 | 0 | 25 | 0 |
| 18 | 0 | 75 | 0 | 0 |
| 19 | 0 | 25 | 0 | 0 |
| 20 | 50 | 25 | 0 | 50 |
| 21 | 25 | 0 | 25 | 0 |
| 22 | 25 | 0 | 0 | 25 |
| 23 | 0 | 25 | 0 | 0 |
| 24 | 0 | 25 | 0 | 0 |
| 25 | 0 | 25 | 0 | 25 |
| 26 | 0 | 25 | 25 | 25 |
| 27 | 0 | 25 | 0 | 50 |
| 28 | 25 | 25 | 25 | 25 |
| 29 | 25 | 0 | 0 | 25 |
| 30 | 0 | 25 | 0 | 0 |
| **Average (95%CI)** | **15.8 (9.9, 21.8)** | **15.8 (9.0, 22.7)** | **7.5 (2.7, 12.3)** | **17.5 (10.4, 24.6)** |

| **Supplementary Table 18.** Misclassification rates (percentage) on the back pain and pain-free clusters in the sub-domain analysis on spinal tissues only. | | | | |
| --- | --- | --- | --- | --- |
| **Run** | **SVM** | **Naïve-Bayes** | **kNN** | **Random Forest** |
| 1 | 25 | 25 | 25 | 25 |
| 2 | 25 | 25 | 37.5 | 0 |
| 3 | 12.5 | 12.5 | 12.5 | 12.5 |
| 4 | 50 | 25 | 37.5 | 12.5 |
| 5 | 12.5 | 50 | 37.5 | 12.5 |
| 6 | 37.5 | 37.5 | 37.5 | 12.5 |
| 7 | 25 | 37.5 | 62.5 | 50 |
| 8 | 12.5 | 25 | 75 | 50 |
| 9 | 25 | 12.5 | 50 | 62.5 |
| 10 | 62.5 | 37.5 | 25 | 37.5 |
| 11 | 25 | 25 | 25 | 12.5 |
| 12 | 37.5 | 37.5 | 25 | 37.5 |
| 13 | 25 | 37.5 | 50 | 25 |
| 14 | 12.5 | 37.5 | 50 | 25 |
| 15 | 0 | 12.5 | 50 | 12.5 |
| 16 | 37.5 | 37.5 | 50 | 62.5 |
| 17 | 0 | 25 | 25 | 12.5 |
| 18 | 12.5 | 37.5 | 37.5 | 25 |
| 19 | 25 | 50 | 37.5 | 0 |
| 20 | 50 | 12.5 | 25 | 12.5 |
| 21 | 37.5 | 50 | 50 | 25 |
| 22 | 12.5 | 37.5 | 37.5 | 25 |
| 23 | 37.5 | 50 | 50 | 50 |
| 24 | 25 | 25 | 37.5 | 37.5 |
| 25 | 37.5 | 25 | 25 | 37.5 |
| 26 | 25 | 62.5 | 50 | 37.5 |
| 27 | 12.5 | 37.5 | 37.5 | 37.5 |
| 28 | 37.5 | 37.5 | 25 | 25 |
| 29 | 37.5 | 37.5 | 25 | 37.5 |
| 30 | 25 | 12.5 | 12.5 | 37.5 |
| **Average (95%CI)** | **26.7 (21.4, 31.9)** | **32.5 (27.9, 37.1)** | **37.5 (32.4, 42.6)** | **28.3 (22.4, 34.2)** |

| **Supplementary Table 19.** Descriptive statistics of pain-free and derived CLBP sub-groups from the sub-domain analysis on the spinal tissues. | | | | | | |
| --- | --- | --- | --- | --- | --- | --- |
|  | **Pain-Free (n=21)** | **CLBP Sub-Group 1 (n=11)** | **p-value** | **CLBP Sub-Group 2 (n=10)** | **p-value** | **p-value between CLBP groups** |
| **Ages (years)** | 35.6 (11.1) | 43.1 (8.6) | 0.102 | 27.0 (6.8) | 0.061 | **0.001** |
| **Sex (Male/Female – n)** | 10/11 | 4/7 | - | 6/4 | - | 0.556 |
| **Height (cm)** | 172.9 (9.1) | 170.4 (9.7) | 0.755 | 178.0 (9.8) | 0.352 | 0.170 |
| **Body mass index (kg/cm^2^)** | 25.3 (6.1) | 29.1 (5.6) | 0.159 | 25.1 (3.5) | 0.997 | 0.233 |
| **Pain Intensity (0-100)**  *Current*  *Last-week average*  *Last-week worst* | -  -  - | 37.2 (22.8)  42.2 (19.0)  64.1 (22.0) | -  -  - | 26.0 (15.1)  34.2 (16.0)  49.4 (18.0) | -  -  - | 0.164  0.327  0.070 |
| **Oswestry Disability Index (0-100)** | - | 28.7 (11.8) | - | 16.4 (5.2) | - | **0.004** |
| **Number of pain sites over the last 12 months** | 1.4 (1.4) | 5.9 (2.1) | **<0.001** | 5.1 (2.7) | **<0.001** | 0.621 |
| **Central Sensitisation Inventory (0-100)** | 17.1 (10.3) | 39.8 (14.8) | **<0.001** | 33.5 (13.1) | **0.003** | 0.472 |
| **Satisfaction in Social Roles (8-40)*** | 38.1 (3.8) | 29.0 (10.3) | **0.001** | 36.1 (4.1) | 0.695 | **0.032** |
| **Depressive Symptoms (8-40)** | 8.8 (1.3) | 12.6 (5.0) | 0.064 | 11.6 (7.2) | 0.221 | 0.873 |
| **Maximal Extension Strength (kg)** | 68.7 (16.2) | 40.7 (15.3) | **<0.001** | 71.5 (12.4) | 0.886 | **<0.001** |
| **Average lumbar pressure-pain thresholds (kg/cm^2^)** | 9.1 (2.2) | 7.9 (3.0) | 0.383 | 6.9 (2.2) | 0.061 | 0.631 |
| **General Self-Efficacy (10-50)*** | 33.1 (5.4) | 36.9 (9.7) | **0.020** | 42.0 (5.9) | 0.706 | 0.216 |
| **Symptoms of Anxiety (8-40)** | 11.5 (4.4) | 16.6 (6.9) | 0.067 | 14.3 (7.6) | 0.453 | 0.645 |
| **Cognitive Function (8-40)** | 36.2 (4.4) | 31.8 (8.2) | 0.177 | 32.2 (8.0) | 0.254 | 0.990 |
| **Average Lumbar T2-time (ms)** | 103.4 (13.5) | 87.2 (4.8) | **<0.001** | 104.7 (8.3) | 0.946 | **0.002** |
| Data are reported as mean and standard deviation unless otherwise specified.  *Higher values are better.  **Results of chi-square test between all groups. | | | | | | |

| **Supplementary Table 20.** Cluster validity methods to determine appropriate cluster number for all variables in the sub-domain analyses on the nervous system only. | | | |
| --- | --- | --- | --- |
| **Cluster Number** | **Calinski-Harabasz*** | **Davies-Bouldin**** | **Sillhouette*** |
| *2-Variables* | | | |
| K=2 | 14.6 | 1.07 | 0.53 |
| K=3 | 18.0 | 0.86 | 0.55 |
| K=4 | 20.3 | 0.79 | 0.57 |
| K=5 | **29.4** | **0.61** | **0.70** |
| K=6 | 26.2 | 0.74 | 0.56 |
| *Higher values for Calinski-Harabasz and Silhouette values indicate the optimal cluster number. **Lower values for Davies-Bouldin values indicate the optimal class number. | | | |

| **Supplementary Table 21.** Average within- and between-cluster distances on a normalised 0-1 scale for the sub-domain analysis on the nervous system only. | | | | | |
| --- | --- | --- | --- | --- | --- |
|  | Cluster 1 | Cluster 2 | Cluster 3 | Cluster 4 | Cluster 5 |
| *Within-Cluster Distances* | | | | | |
| Distance | 0.19 | 0.09 | 0.12 | 0.15 | 0.10 |
| *Between-Cluster Distances (Discrimination Value)* | | | | | |
| Cluster 1 | 0 | 1.29 (-2.3) | 2.13 (-4.0) | 3.04 (-5.7) | 4.12 (-7.9) |
| Cluster 2 | 1.29 (-2.3) | 0 | 1.42 (-2.6) | 2.05 (-3.9) | 3.05 (-5.9) |
| Cluster 3 | 2.13 (-4.0) | 1.42 (-2.6) | 0 | 1.16 (-1.9) | 2.14 (-4.1) |
| Cluster 4 | 3.04 (-5.7) | 2.05 (-3.9) | 1.16 (-1.9) | 0 | 1.12 (-2.0) |
| Cluster 5 | 4.12 (-7.9) | 3.05 (-5.9) | 2.14 (-4.1) | 1.12 (-2.0) | 0 |

| **Supplementary Table 22.** Misclassification rates (percentage) on the back pain clusters in the sub‑domain analysis on the nervous system only. | | | | |
| --- | --- | --- | --- | --- |
| **Run** | **SVM** | **Naïve-Bayes** | **kNN** | **Random Forest** |
| 1 | 50 | 75 | 25 | 50 |
| 2 | 25 | 50 | 50 | 50 |
| 3 | 25 | 50 | 0 | 50 |
| 4 | 0 | 50 | 25 | 25 |
| 5 | 25 | 25 | 25 | 25 |
| 6 | 0 | 0 | 50 | 25 |
| 7 | 0 | 50 | 50 | 25 |
| 8 | 0 | 50 | 50 | 25 |
| 9 | 0 | 25 | 50 | 50 |
| 10 | 50 | 75 | 25 | 25 |
| 11 | 50 | 25 | 75 | 50 |
| 12 | 50 | 25 | 75 | 75 |
| 13 | 50 | 75 | 0 | 50 |
| 14 | 25 | 75 | 25 | 25 |
| 15 | 50 | 50 | 25 | 50 |
| 16 | 25 | 75 | 0 | 50 |
| 17 | 25 | 50 | 25 | 25 |
| 18 | 25 | 50 | 50 | 25 |
| 19 | 0 | 75 | 50 | 25 |
| 20 | 50 | 25 | 50 | 25 |
| 21 | 25 | 50 | 50 | 25 |
| 22 | 0 | 75 | 25 | 50 |
| 23 | 0 | 75 | 50 | 25 |
| 24 | 0 | 50 | 75 | 50 |
| 25 | 25 | 75 | 0 | 50 |
| 26 | 0 | 50 | 25 | 50 |
| 27 | 25 | 75 | 50 | 25 |
| 28 | 25 | 50 | 25 | 25 |
| 29 | 0 | 50 | 25 | 25 |
| 30 | 25 | 25 | 25 | 25 |
| **Average (95%CI)** | **21.7 (14.7, 28.6)** | **51.7 (44.3, 59.1)** | **35.8 (28.2, 43.5)** | **36.7 (31.6, 41.8)** |

| **Supplementary Table 23.** Misclassification rates (percentage) on the back pain and pain-free clusters in the sub-domain analysis on the nervous system only. | | | | |
| --- | --- | --- | --- | --- |
| **Run** | **SVM** | **Naïve-Bayes** | **kNN** | **Random Forest** |
| 1 | 25 | 25 | 37.5 | 25 |
| 2 | 37.5 | 37.5 | 75 | 25 |
| 3 | 37.5 | 37.5 | 37.5 | 37.5 |
| 4 | 12.5 | 25 | 50 | 25 |
| 5 | 50 | 37.5 | 87.5 | 25 |
| 6 | 25 | 12.5 | 50 | 37.5 |
| 7 | 75 | 62.5 | 50 | 25 |
| 8 | 25 | 50 | 75 | 37.5 |
| 9 | 25 | 50 | 37.5 | 62.5 |
| 10 | 25 | 87.5 | 62.5 | 12.5 |
| 11 | 12.5 | 50 | 75 | 25 |
| 12 | 12.5 | 25 | 62.5 | 37.5 |
| 13 | 37.5 | 50 | 62.5 | 25 |
| 14 | 37.5 | 25 | 62.5 | 25 |
| 15 | 25 | 62.5 | 37.5 | 25 |
| 16 | 12.5 | 50 | 62.5 | 37.5 |
| 17 | 50 | 25 | 50 | 50 |
| 18 | 37.5 | 50 | 75 | 37.5 |
| 19 | 37.5 | 37.5 | 50 | 37.5 |
| 20 | 50 | 75 | 37.5 | 50 |
| 21 | 12.5 | 37.5 | 37.5 | 25 |
| 22 | 25 | 37.5 | 37.5 | 25 |
| 23 | 50 | 50 | 50 | 37.5 |
| 24 | 12.5 | 50 | 37.5 | 12.5 |
| 25 | 12.5 | 37.5 | 12.5 | 37.5 |
| 26 | 12.5 | 75 | 37.5 | 37.5 |
| 27 | 37.5 | 37.5 | 50 | 37.5 |
| 28 | 12.5 | 25 | 37.5 | 12.5 |
| 29 | 37.5 | 37.5 | 25 | 25 |
| 30 | 50 | 37.5 | 50 | 12.5 |
| **Average (95%CI)** | **30.4 (24.7, 36.1)** | **43.3 (37.3, 49.4)** | **50.4 (44.4, 56.5)** | **30.8 (26.6, 35.0)** |

| **Supplementary Table 24.** Descriptive statistics of pain-free and derived CLBP sub-groups from the sub-domain analysis on the nervous system domain. | | | | | | | | | | |  |
| --- | --- | --- | --- | --- | --- | --- | --- | --- | --- | --- | --- |
|  | **Pain-Free (n=21)** | **CLBP Sub-Group 1 (n=3)** | **p-value** | **CLBP Sub-Group 2 (n=4)** | **p-value** | **CLBP Sub-Group 3 (n=4)** | **p-value** | **CLBP Sub-Group 4 (n=8)** | **p-value** | **CLBP Sub-Group 5 (n=2)** | **p-value** |
| **Ages (years)** | 35.6 (11.1) | 35.6 (6.1) | 1.00 | 43.3 (9.3) | 0.786 | 40.3 (12.8) | 0.968 | 28.6 (8.9) | 0.630 | 37.0 (18.4) | 1.00 |
| **Sex (Male/Female – n)** | 10/11 | 3/0 | - | 2/2 | - | 0/4 | - | 4/4 | - | 1/1 | 0.223** |
| **Height (cm)** | 172.9 (9.1) | 173.8 (6.5) | 1.00 | 174.8 (11.6) | 0.999 | 165.3 (3.7) | 0.702 | 177.3 (12.4) | 0.876 | 176.6 (9.3) | 0.995 |
| **Body mass index (kg/cm^2^)** | 25.3 (6.1) | 27.2 (5.9) | 0.993 | 28.8 (4.5) | 0.851 | 30.9 (7.2) | 0.446 | 25.4 (3.8) | 1.00 | 23.7 (1.7) | 0.999 |
| **Pain Intensity (0-100)**  *Current*  *Last-week average*  *Last-week worst* | -  -  - | 31.7 (15.6)  36.3 (2.5)  64.7 (10.3) | -  -  - | 12 (8.9)  23.3 (13.9)  53.3 (26.2) | -  -  - | 42.8 (18.1)  52.3 (17.8)  67.8 (25.5) | -  -  - | 35.0 (23.8)  36.8 (19.6)  50.8 (22.8) | -  -  - | 37.5 (2.1)  48.5 (2.1)  57.5 (2.1) | -  -  - |
| **Oswestry Disability Index (0-100)** | - | 18.0 (6.9) | - | 20.5 (7.2) | - | 32.5 (18.0) | **-** | 19.8 (8.5) | **-** | 28.0 (11.3) | **-** |
| **Number of pain sites over the last 12 months** | 1.4 (1.4) | 5.3 (4.1) | **0.023** | 5.0 (1.4) | **0.017** | 6.5 (1.0) | **<0.001** | 4.8 (2.7) | **0.002** | 8.0 (1.4) | **<0.001** |
| **Central Sensitisation Inventory (0-100)** | 17.1 (10.3) | 20.3 (8.5) | 0.990 | 26.0 (4.8) | 0.436 | 56.3 (5.3) | **<0.001** | 34.8 (6.4) | **<0.001** | 52.5 (6.4) | **<0.001** |
| **Satisfaction in Social Roles (8-40)*** | 38.1 (3.8) | 37.6 (4.0) | 0.999 | 33.5 (11.1) | 0.780 | 25.8 (10.2) | **0.013** | 33.9 (5.9) | 0.621 | 29.5 (14.9) | 0.473 |
| **Depressive Symptoms (8-40)** | 8.8 (1.3) | 10.0 (2.0) | 0.994 | 9.8 (2.4) | 0.997 | 18.0 (9.9) | **0.001** | 10.4 (3.4) | 0.266 | 15.0 (9.9) | 0.999 |
| **Maximal Extension Strength (kg)** | 68.7 (16.2) | 67.7 (35.1) | 0.999 | 38.1 (16.0) | **0.042** | 54.3 (5.2) | 0.699 | 57.3 (22.0) | 0.665 | 65.7 (8.1) | 0.999 |
| **Average lumbar pressure-pain thresholds (kg/cm^2^)** | 9.1 (2.2) | 4.4 (1.3) | **0.001** | 10.7 (0.6) | 0.477 | 4.4 (0.7) | **<0.001** | 7.6 (0.9) | 0.313 | 10.4 (0.5) | 0.894 |
| **General Self-Efficacy (10-50)*** | 44.1 (5.4) | 40.3 (5.1) | 0.932 | 43.3 (3.3) | 0.999 | 33.0 (10.4) | **0.036** | 42.4 (6.9) | 0.986 | 30.5 (13.4) | 0.072 |
| **Symptoms of Anxiety (8-40)** | 11.5 (4.4) | 8.3 (0.6) | 0.919 | 11.2 (3.0) | 0.999 | 22.2 (7.1) | **0.007** | 16.3 (5.3) | 0.274 | 18.5 (14.9) | 0.477 |
| **Cognitive Function (8-40)** | 36.2 (4.4) | 39.3 (1.2) | 0.916 | 38.8 (2.5) | 0.939 | 25.3 (6.7) | **0.004** | 31.5 (7.1) | 0.258 | 23.0 (7.1) | **0.015** |
| **Average Lumbar T2-time (ms)** | 103.4 (13.5) | 102.0 (12.6) | 0.999 | 84.8 (3.9) | 0.081 | 93.9 (9.2) | 0.706 | 99.3 (12.0) | 0.963 | 95.8 (11.3) | 0.956 |
| Data are reported as mean and standard deviation unless otherwise specified.  *Higher values are better.  **Results of chi-square test between all groups. | | | | | | | | | | | |

**SUPPLEMENTARY FIGURES**


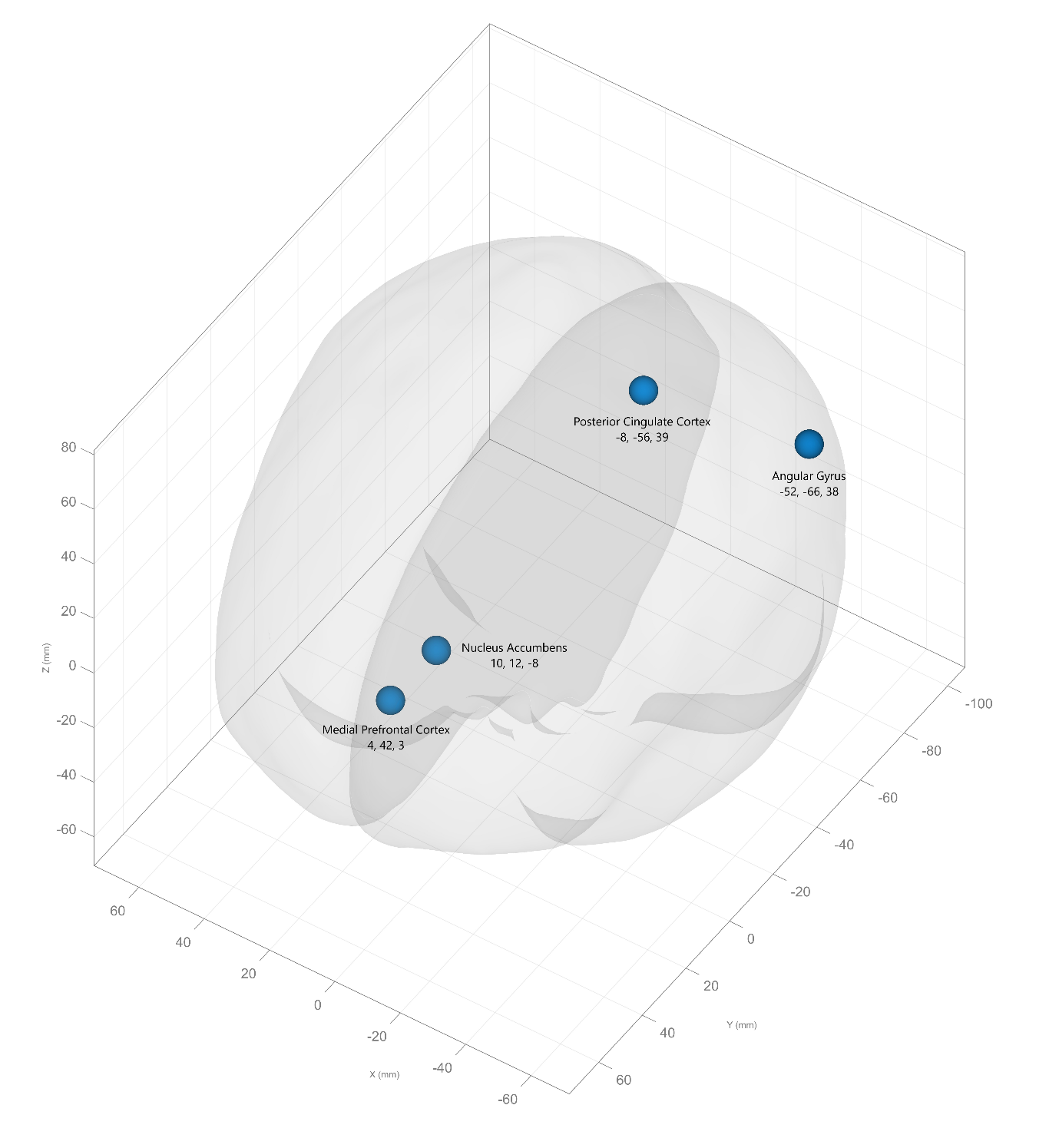


**Supplementary Figure 1.** Location of spheres for the medial prefrontal cortex, nucleus accumbens, posterior cingulate cortex, and angular gyrus used in resting state functional connectivity analyses.


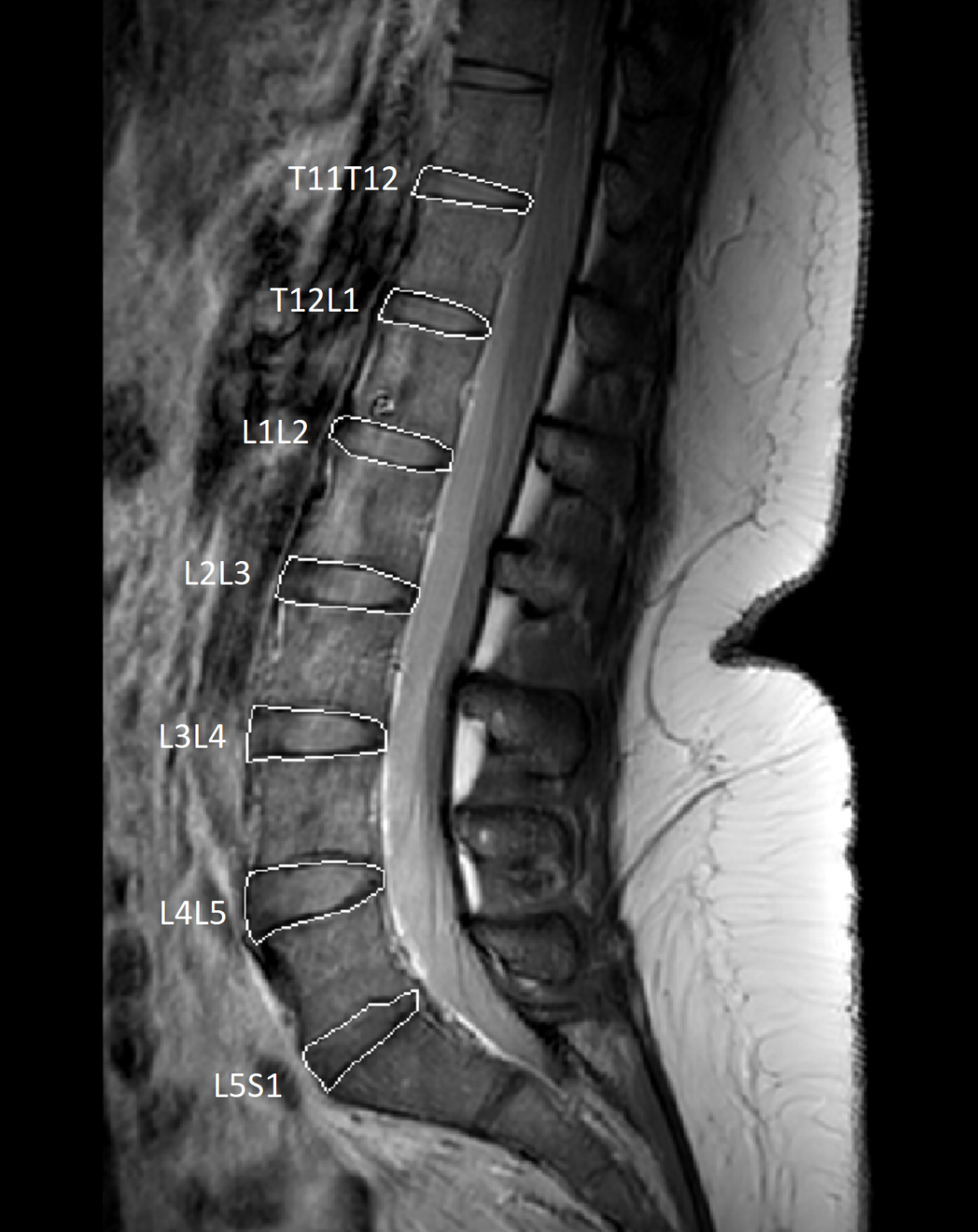


**Supplementary Figure 2.** Tracing of the intervertebral discs from L5/S1 level to the T11/T12 level.


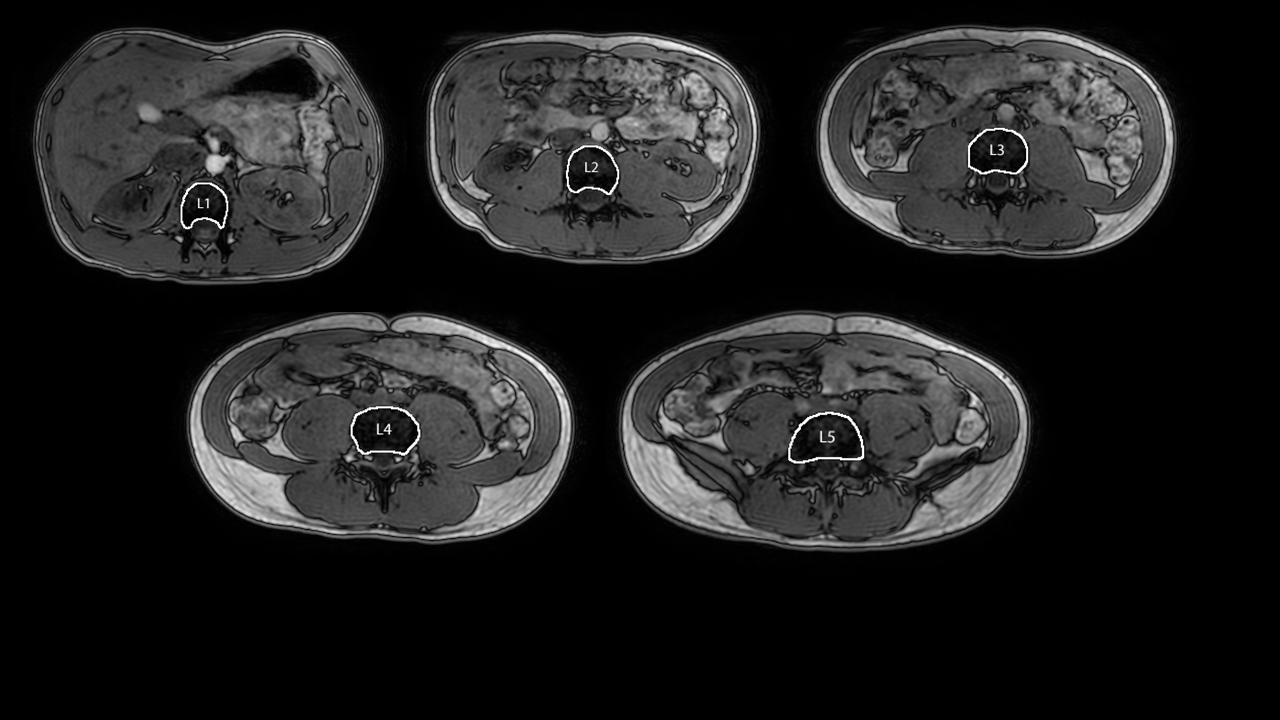


**Supplementary Figure 3.** Tracing of the lumbar vertebrae on the axial DIXON out of phase image.


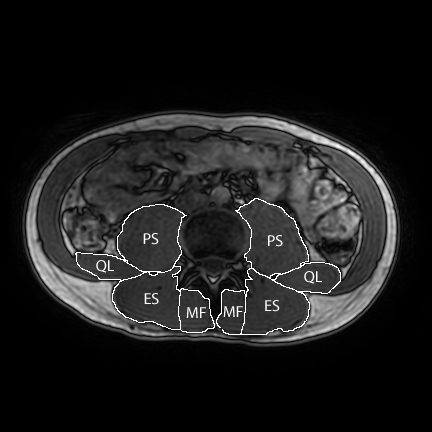


**Supplementary Figure 4.** Tracing of the multifidus (MF), erector spinae (ES), psoas major (PS), and quadratus lumborum (PS) at the top of the L4 vertebrae on the axial DIXON out of phase image.
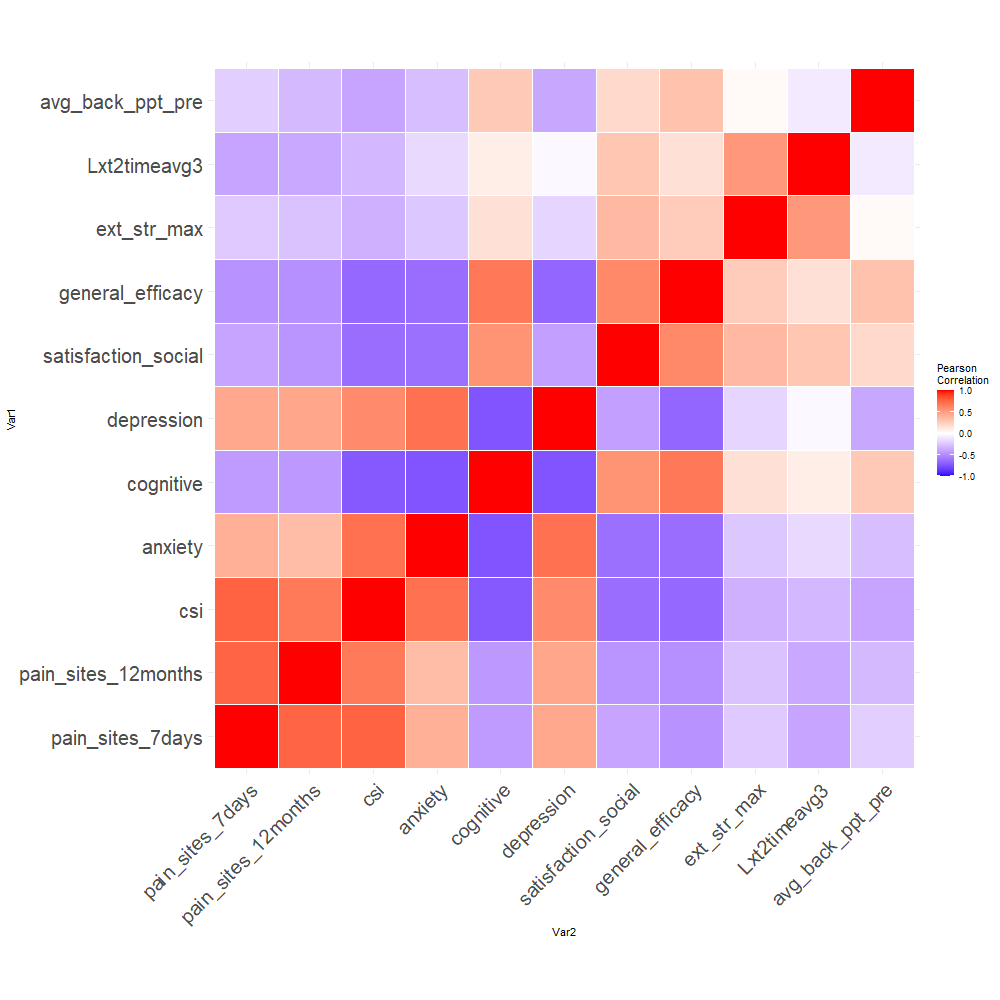


**Supplementary Figure 5.** Heat map of Pearson’s correlation coefficients between significant variables in the primary analyses.


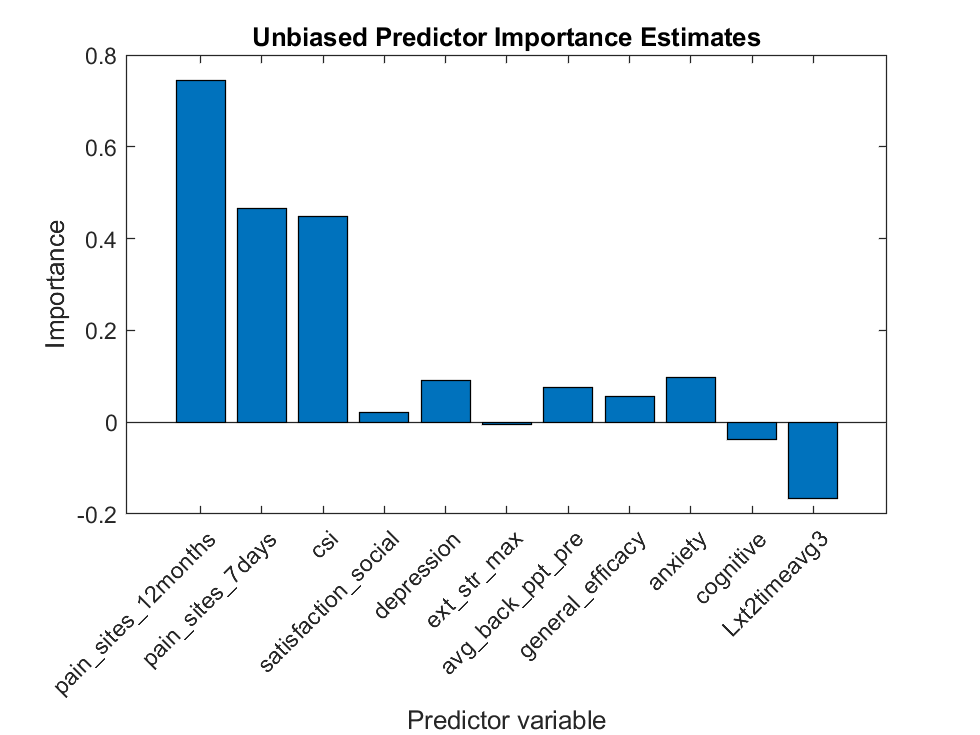


**Supplementary Figure 6.** Random Forest variable predictor for feature weighting for the primary analysis.


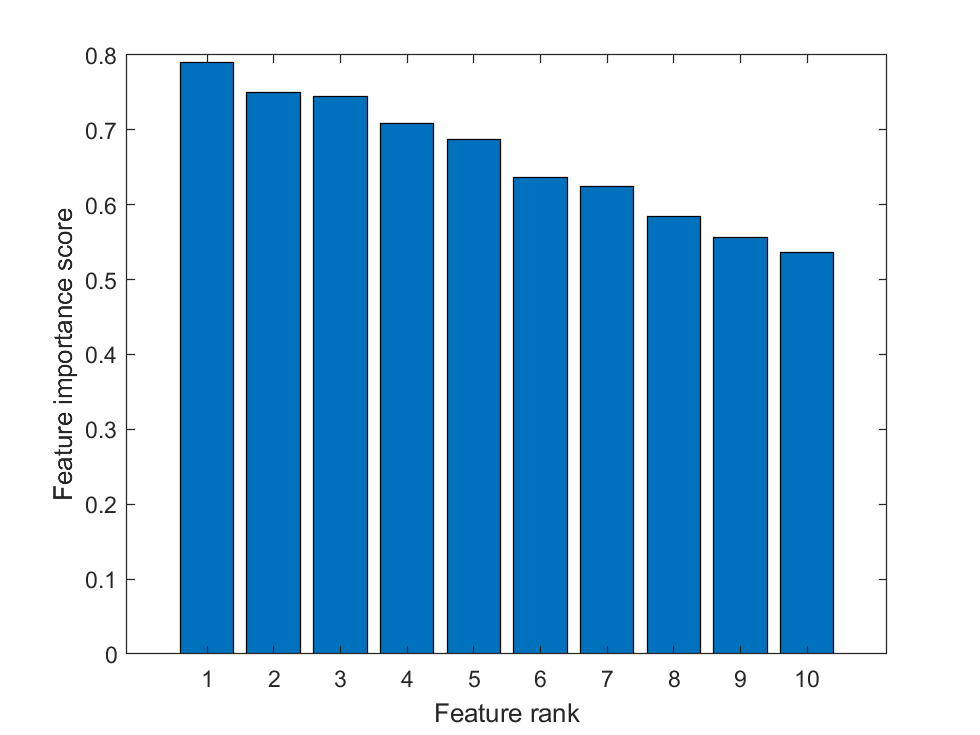


**Supplementary Figure 7.** Laplacian scores indicated the feature importance in the CLBP only space. In order of importance, the features were, (1) cognitive function, (2) depressive symptoms, (3) anxiety symptoms, (4) general self-efficacy, (5) satisfaction in social roles, (6) central sensitisation, (7) pain site within the last 12 months, (8) average lumbar pressure-pain thresholds, (9) average lumbar T2‑time, (1) maximal extension strength.


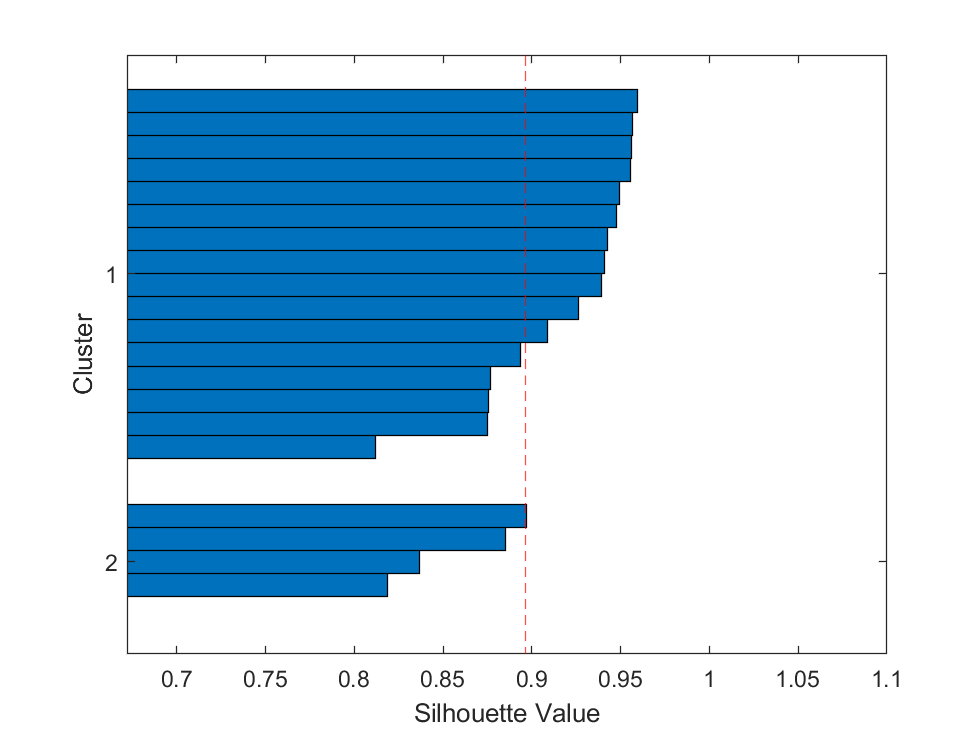


**Supplementary Figure 8.** Silhouette values for sub-groups derived from fuzzy c-means clustering. The Silhouette value is 0.89.


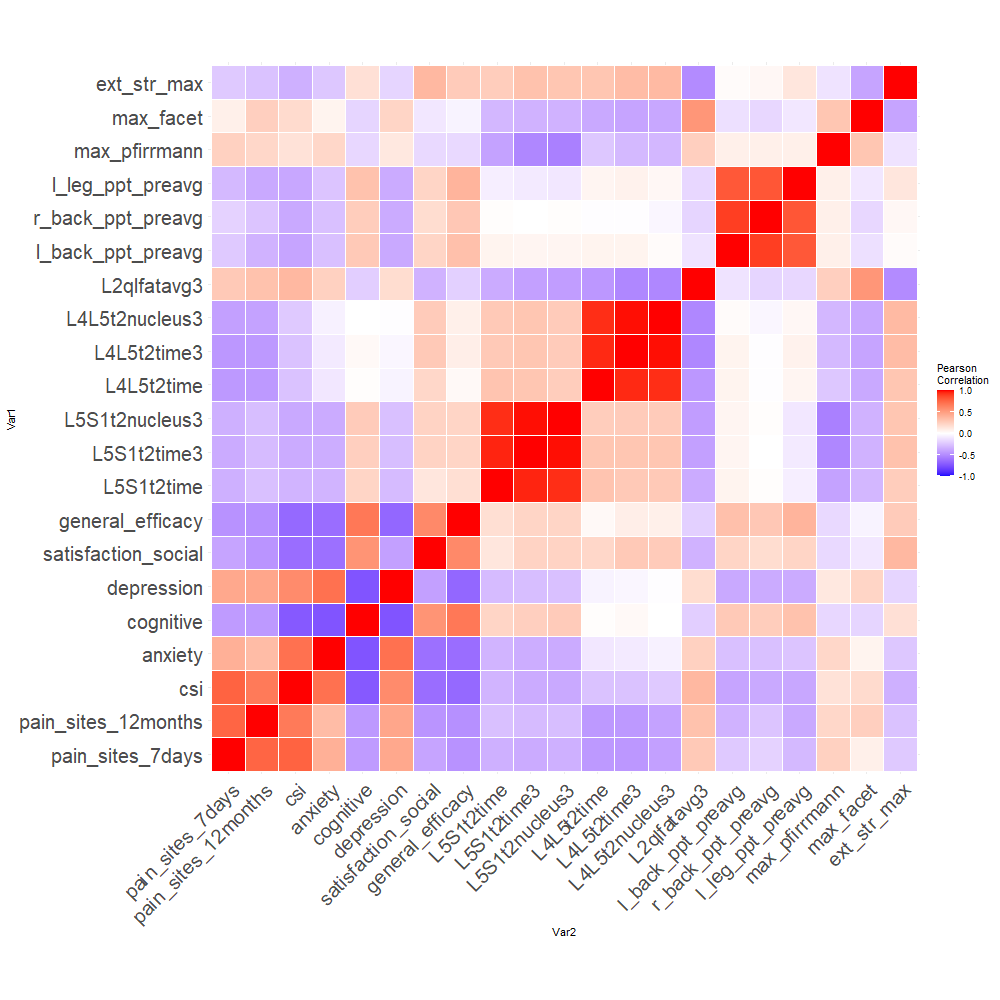


**Supplementary Figure 9.** Heat map of Pearson’s correlation coefficients between significant variables in the secondary analyses.


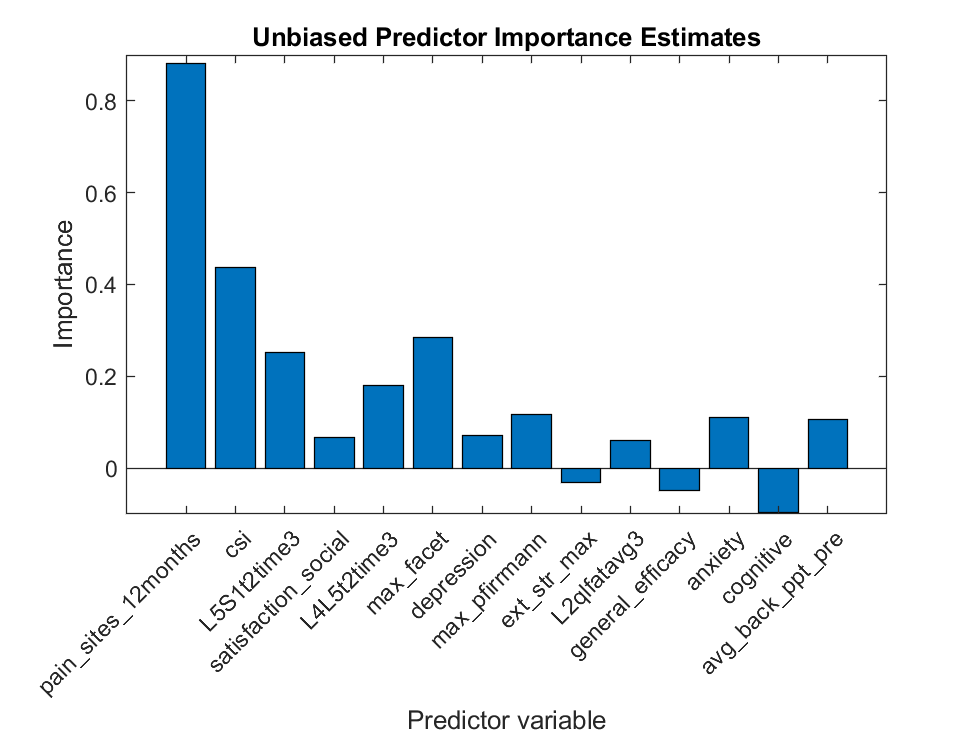


**Supplementary Figure 10.** Random Forest variable predictor for feature weighting for the secondary analysis.


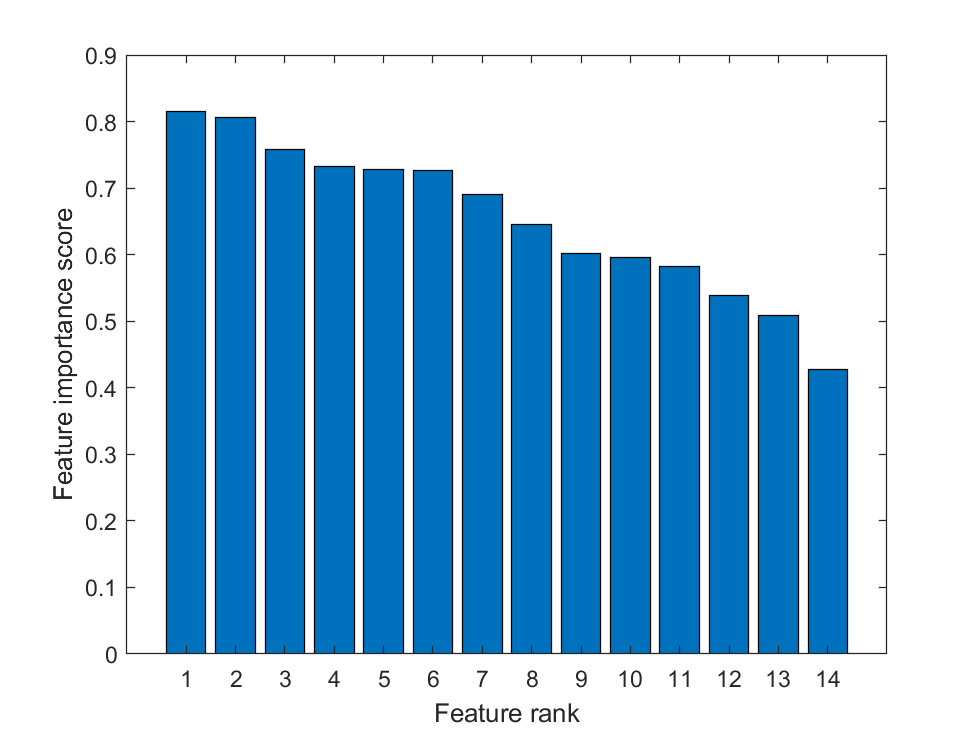


**Supplementary Figure 11.** Laplacian scores indicated the feature importance in the CLBP only space. In order of importance, the features were, (1) cognitive function, (2) depressive symptoms, (3) general self-efficacy, (4) maximum facet joint grading, (5) anxiety symptoms, (6) satisfaction in social roles, (7) central sensitisation inventory, (8) maximal extension strength, (9) L2 quadratus lumborum fat fraction, (10) number of pain sites over the last 12 months, (11) average back pressure-pain thresholds, (12) L5S1 T2-time, (13) L4L5 T2-time and (14) maximal Pfirrmann grade.


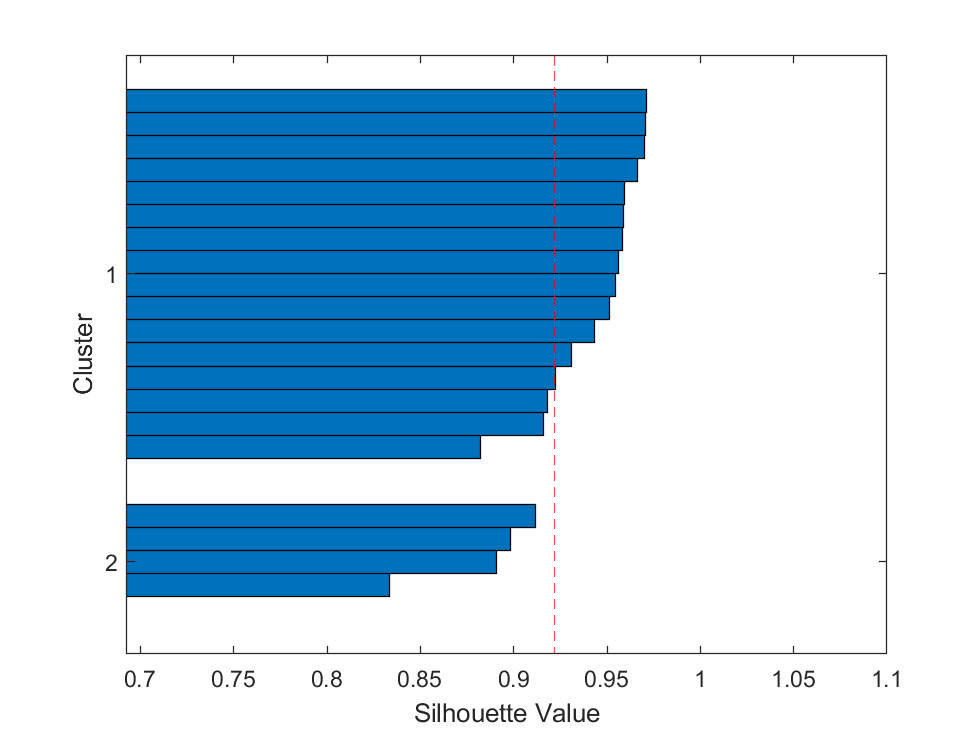


**Supplementary Figure 12.** Silhouette values for sub-groups derived from fuzzy c-means clustering for the secondary analyses. The Silhouette value is 0.92.


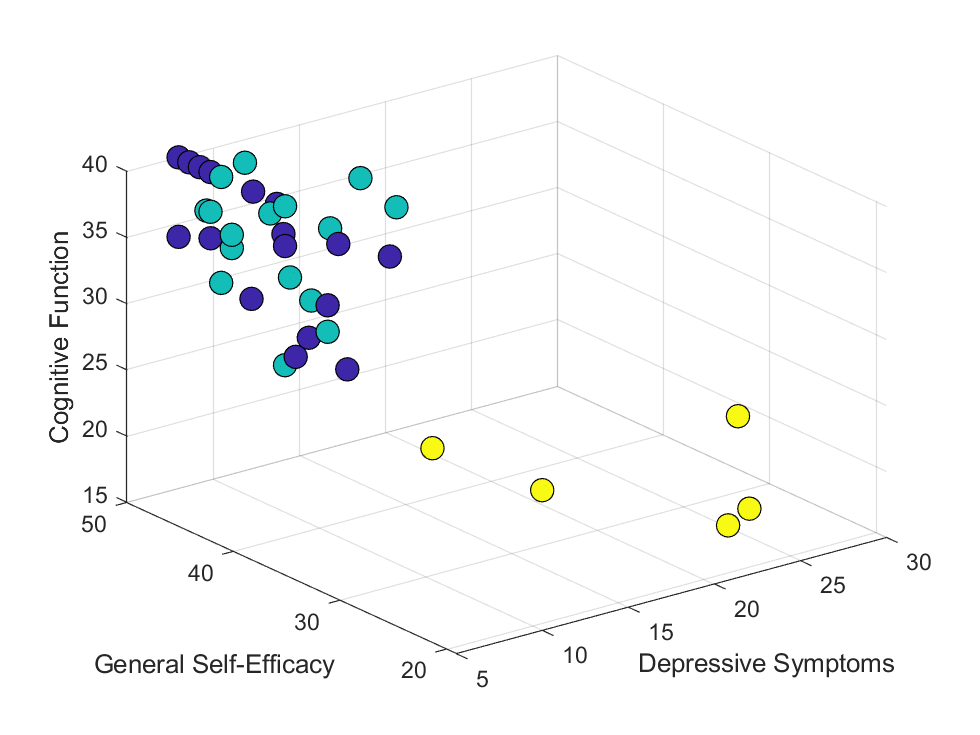


**Supplementary Figure 13.** Three-dimensional scatter plot of variables used to derive sub-groups in the secondary analyses.


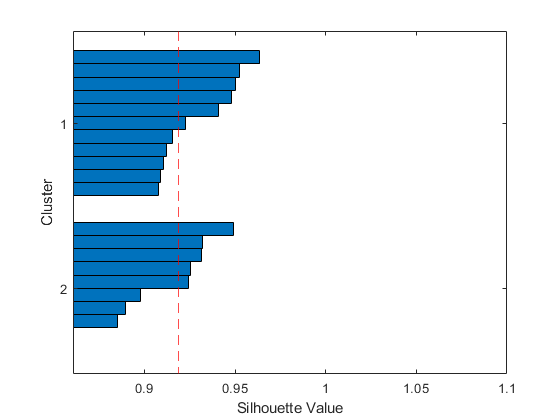


**Supplementary Figure 14.** Silhouette values for sub-groups derived from fuzzy c-means clustering for the sub-domain analysis on the spinal tissues only. The Silhouette value is 0.92.


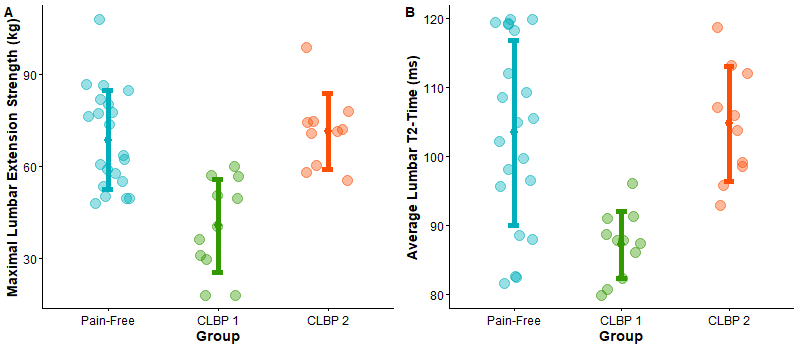


**Supplementary Figure 15.** Mean and standard deviations, plus individual data points, for sub-groups derived on spinal tissue measures on maximal lumbar extension strength and average lumbar T2-time.


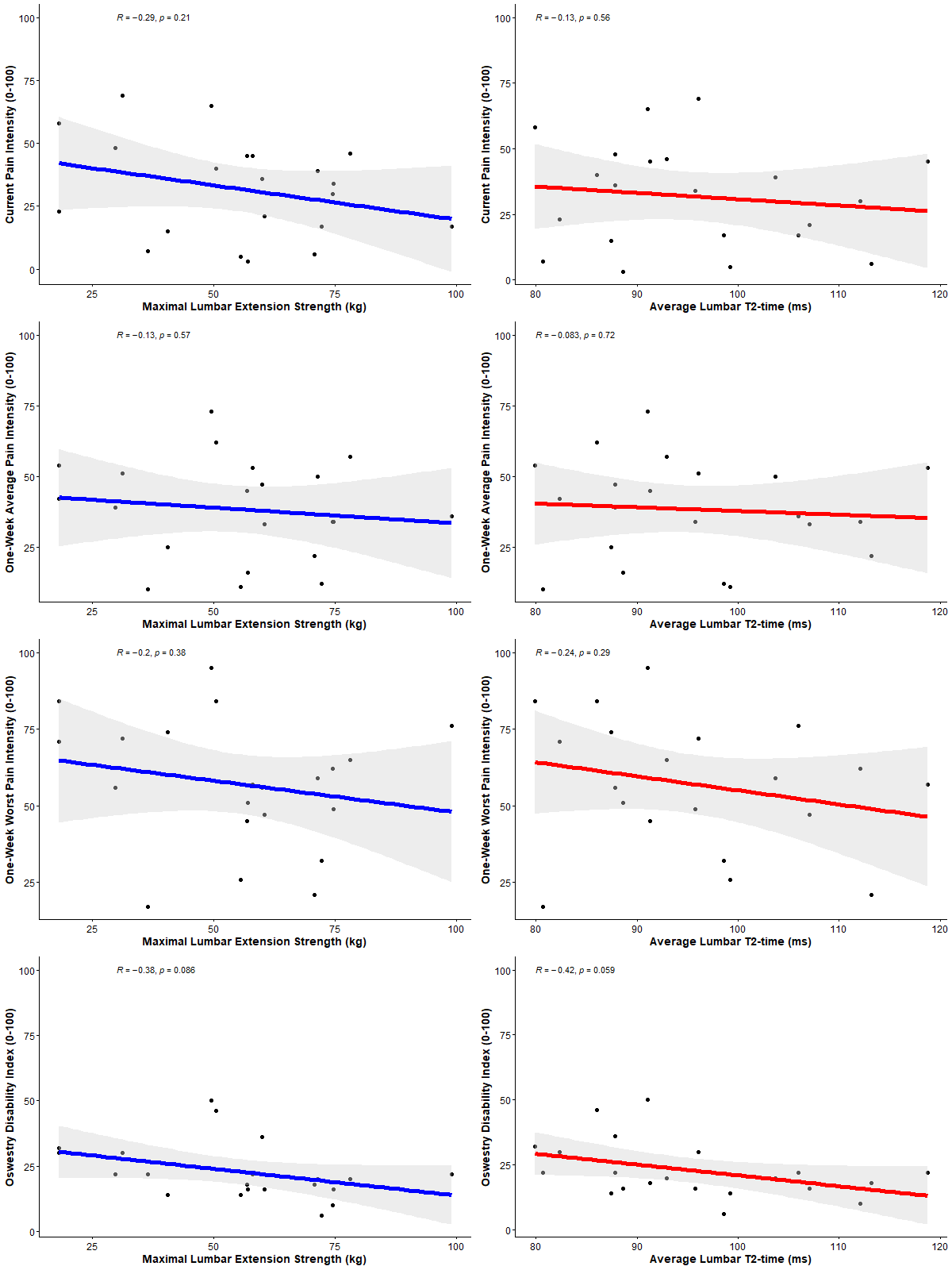


**Supplementary Figure 16.** Scatter plots indicated the Pearson’s correlation coefficient and 95% confidence interval between variables deriving the CLBP sub-groups in the spinal tissue analyses and clinical outcomes of pain intensity and disability.


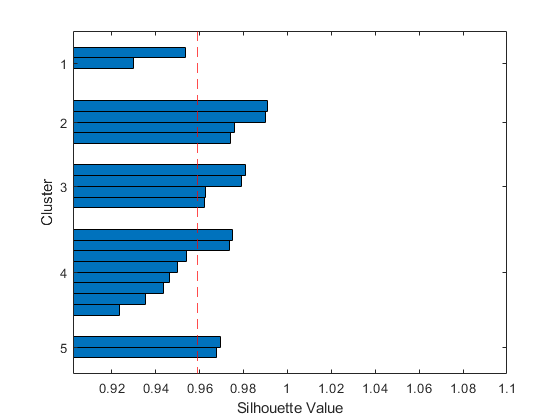


**Supplementary Figure 17.** Silhouette values for sub-groups derived from fuzzy c-means clustering for the sub-domain analysis on the nervous system only. The Silhouette value is 0.96.


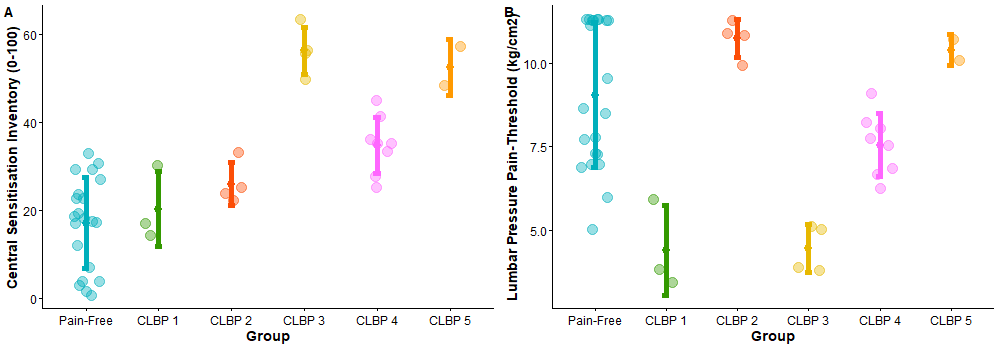


**Supplementary Figure 18.** Mean and standard deviations, plus individual data points, for sub-groups derived on nervous system measures of central sensitisation and lumbar pressure-pain thresholds.


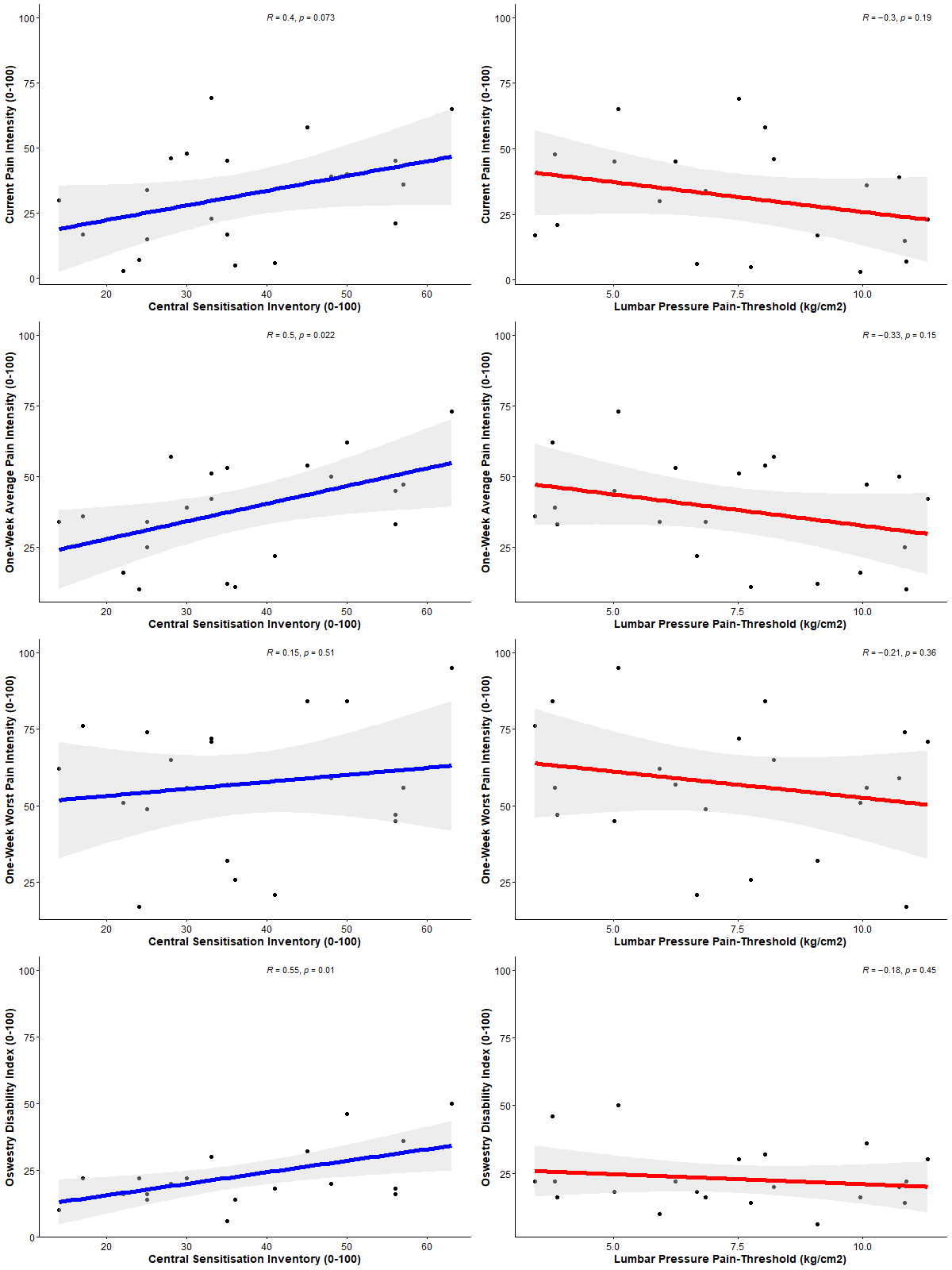


**Supplementary Figure 19.** Scatter plots indicated the Pearson’s correlation coefficient and 95% confidence interval between variables deriving the CLBP sub-groups in the nervous system analyses and clinical outcomes of pain intensity and disability.

**REFERENCES**

1. Pfirrmann CW, Metzdorf A, Zanetti M, Hodler J, Boos N. Magnetic resonance classification of lumbar intervertebral disc degeneration. *Spine (Phila Pa 1976)*. 2001;26(17):1873-1878. doi:10.1097/00007632-200109010-00011

2. Weishaupt D, Zanetti M, Boos N, Hodler J. MR imaging and CT in osteoarthritis of the lumbar facet joints. *Skeletal Radiol*. 1999;28(4):215-219. doi:10.1007/s002560050503

3. Dunn AJ, Campbell RSD, Mayor PE, Rees D. Radiological findings and healing patterns of incomplete stress fractures of the pars interarticularis. *Skeletal Radiol*. 2008;37(5):443-450. doi:10.1007/s00256-008-0449-0

4. Jensen MC, Brant-Zawadzki MN, Obuchowski N, Modic MT, Malkasian D, Ross JS. Magnetic resonance imaging of the lumbar spine in people without back pain. *N Engl J Med*. 1994;331(2):69-73. doi:10.1056/NEJM199407143310201

5. Videman T, Battié MC, Gill K, Manninen H, Gibbons LE, Fisher LD. Magnetic resonance imaging findings and their relationships in the thoracic and lumbar spine. Insights into the etiopathogenesis of spinal degeneration. *Spine (Phila Pa 1976)*. 1995;20(8):928-935. doi:10.1097/00007632-199504150-00009
